# A virtual pilot optimization trial for African American/Black and Latino persons with non-suppressed HIV viral load grounded in motivational interviewing and behavioral economics

**DOI:** 10.3389/fpubh.2023.1167104

**Published:** 2023-05-10

**Authors:** Prema Filippone, Samantha Serrano, Marya Gwadz, Charles M. Cleland, Robin Freeman, Sebastian Linnemayr, Sabrina R. Cluesman, Stephanie Campos, Corey Rosmarin-DeStefano, Brianna Amos, Khadija Israel

**Affiliations:** ^1^Intervention Innovations Team Lab (IIT-Lab), New York University Silver School of Social Work, New York, NY, United States; ^2^Center for Drug Use and HIV Research, School of Global Public Health, New York University, New York, NY, United States; ^3^Division of Biostatistics, Department of Population Health, New York University School of Medicine, New York, NY, United States; ^4^Independent Consultant, New York, NY, United States; ^5^Pardee RAND Graduate School, Santa Monica, CA, United States; ^6^North Jersey Community Research Initiative, Newark, NJ, United States

**Keywords:** HIV care continuum, multiphase optimization strategy (MOST), mixed methods, motivational interviewing, behavioral economics, low-touch, HIV viral suppression, financial incentive

## Abstract

**Introduction:**

Virtual and low-touch behavioral interventions are needed for African American/Black and Latino persons living with HIV (PLWH) with barriers to HIV viral suppression, particularly during COVID-19. Guided by the multiphase optimization strategy, we explored three components for PLWH without viral suppression, grounded in motivational interviewing and behavioral economics: (1) motivational interviewing counseling, (2) 21-weeks of automated text messages and quiz questions about HIV management, and (3) financial rewards for viral suppression (lottery prize vs. fixed compensation).

**Methods:**

This pilot optimization trial used sequential explanatory mixed methods to explore the components' feasibility, acceptability, and preliminary evidence of effects using an efficient factorial design. The primary outcome was viral suppression. Participants engaged in baseline and two structured follow-up assessments over an 8-month period, and provided laboratory reports to document HIV viral load. A subset engaged in qualitative interviews. We carried out descriptive quantitative analyses. Then, qualitative data were analyzed using directed content analysis. Data integration used the joint display method.

**Results:**

Participants (*N* = 80) were 49 years old, on average (SD = 9), and 75% were assigned male sex at birth. Most (79%) were African American/Black, and the remainder were Latino. Participants were diagnosed with HIV 20 years previously on average (SD = 9). Overall, components were feasible (>80% attended) and acceptability was satisfactory. A total of 39% (26/66) who provided laboratory reports at follow-up evidenced viral suppression. Findings suggested no components were entirely unsuccessful. The lottery prize compared to fixed compensation was the most promising component level. In qualitative analyses, all components were seen as beneficial to individual wellbeing. The lottery prize appeared more interesting and engaging than fixed compensation. However, structural barriers including financial hardship interfered with abilities to reach viral suppression. The integrated analyses yielded areas of convergence and discrepancy and qualitative findings added depth and context to the quantitative results.

**Conclusions:**

The virtual and/or low-touch behavioral intervention components tested are acceptable and feasible and show enough potential to warrant refinement and testing in future research, particularly the lottery prize. Results must be interpreted in the context of the COVID-19 pandemic.

**Trial registration:**

NCT04518241 (https://clinicaltrials.gov/ct2/show/NCT04518241).

## 1. Introduction

Ending HIV transmission in the United States hinges on preventing new HIV infections (1, 2). This, in turn, requires assisting those already living with HIV to achieve and sustain HIV viral suppression through linkage to HIV primary care, engaging in care regularly, and taking HIV medication with high levels of adherence, a sequence of steps called the HIV care continuum (3–5). As HIV systems of care and treatment improve in the United States, some subpopulations of persons living with HIV (PLWH) benefit from these advances more than others. Although overall rates of HIV care continuum engagement have increased, subgroups of PLWH still experience longstanding and complex barriers to sustained engagement. In particular, racial/ethnic inequities in care continuum engagement and health outcomes are striking. For example, most PLWH are virally suppressed (66%) (1). However, African American/Black and Latino persons living with HIV, who are mainly from socioeconomically disadvantaged backgrounds, experience disproportionately lower rates of engagement along the HIV care continuum compared to White PLWH, including in HIV viral suppression and sustained HIV viral suppression (6–10). Only an estimated 40% of African American/Black and 50% of Latino PLWH sustain HIV viral suppression, compared to 56% of their White counterparts (7). The reasons for these racial/ethnic inequities are multifaceted and include barriers at the structural (e.g., chronic poverty, food insecurity, housing disadvantages, challenges accessing high-quality services), social (e.g., complex stigma, discrimination), cultural (e.g., medical distrust), and individual levels of influence (e.g., substance use and mental health challenges, unstable housing) (11–13). The complexity of barriers that impede access to the HIV care continuum and the fact that racial/ethnic inequities in HIV are serious and persistent signal the need for continued improvement and innovation at all levels, including in behavioral intervention strategies, particularly for subpopulations of PLWH with the greatest impediments to optimal HIV health outcomes.

The COVID-19 pandemic has complicated HIV management and exacerbated existing barriers to HIV care, including for African American/Black and Latino PLWH. Although the Centers for Disease Control and Prevention (CDC) reported expanding measures to support the continuity of HIV care, particularly in high HIV prevalence settings, the COVID-19 pandemic has caused substantive disruptions to engagement along the HIV care continuum (14, 15). Concerns about COVID-19 transmission rates and related social distancing mandates had the effect of constricting access to HIV care by way of reduced clinic hours and provider availability, frequent cancellation of healthcare appointments by both patients and providers, and an increased reliance on telehealth (16). While telehealth visits were useful for PLWH overall, for more disadvantaged PLWH, inadequate access to computer and smartphone technology and internet services precluded such remote visits (17–19). For many PLWH, the stress and emotional toll of social isolation adversely affected mental health, and commonly served as a catalyst for problematic levels of alcohol and drug use, along with increased contact with other people who use drugs, which further impeded engagement along the HIV care continuum (20, 21). Common long-standing structural barriers to HIV management, such as housing instability, food insecurity, and financial hardship, worsened due to the COVID-19 pandemic, related to its economic fallout, and these barriers further obstructed engagement along the care continuum (16, 22). Overall, the COVID-19 pandemic amplified the preexisting racial/ethnic health and economic inequalities so prevalent among African American/Black and Latino PLWH (23–25).

The field of intervention science had focused on virtual and “low-touch” interventions prior to the COVID-19 pandemic, and the COVID-19 pandemic, during which travel on public transportation and in-person contact in professional settings was curtailed, highlighted the importance of such approaches (11, 19). Virtual interventions include those conducted on the phone or a Voice over Internet Protocol (e.g., Webex). Low- and lower-touch interventions consist of those requiring limited staff time for facilitation; for example, because they are technology-based and/or automated (26).

In prior research we developed and explored the acceptability and feasibility of a “lower-touch” intervention for African American/Black and Latino PLWH with non-suppressed HIV viral load. The intervention was called the Silver Community Action Project (S-CAP) and was grounded in motivational interviewing (MI) and behavioral economics and designed to be culturally and structurally relevant by attending to the main barriers to HIV viral suppression experienced by this population, as described above (11, 27). The S-CAP intervention was grounded in work by Linnemayr and colleagues (28–30) and was a multi-component program comprised of a MI counseling session, and 16-weeks of automated text messages (TMs) about HIV management followed by quiz questions, where participants earned points by answering quiz questions (QQs). This aspect of the study was intended to foster engagement in the period during which participants might increase HIV medication use to reach HIV viral suppression, if they wished to and were able to do so. Consistent with behavioral economics (described below), this was followed by a lottery prize, the amount of which was based on viral suppression status (with higher prize amounts for those virally suppressed), number of points earned in the TM component, and chance (max. $275).

The S-CAP intervention was grounded in a conceptual model that integrates critical race theory, harm reduction, and self-determination theory, as described in more detail elsewhere (27, 31). As such, the S-CAP intervention prioritized support of participants' own health decisions and their autonomy overall, reinforced any steps toward positive change, and attended to and sought to resolve structural barriers to HIV management. The S-CAP intervention was designed to be low-touch, in that most activities did not require staff facilitation. It was not designed to be virtual but switched from requiring in-person contact (for enrollment and the MI session) to virtual participation in response to COVID-19 during the study. In a modest pilot study using a pre-test/post-test design and mixed methods, we found the S-CAP intervention was acceptable and feasible, and although it was not powered to examine efficacy, quantitative and qualitative results suggested it had utility and was worth further study (11). The behavior change techniques and approaches that underpin the S-CAP intervention components are incorporated into the present study.

First, MI is an evidence-based directive and collaborative counseling approach for behavior change that elicits participants' values, perspectives, and questions, identifies ambivalence and discrepancies, and corrects misinformation with permission, to thereby foster durable intrinsic motivation and readiness for change (32, 33). MI interventions have been found effective at clinically significant levels across a range of health outcomes (34–36). Moreover, MI has been found highly effective with African American/Black and Latino populations compared to White populations (34). This may be because while MI supports personal decisions and autonomy, such autonomy is often restricted in larger societal and institutional systems in which African American/Black and Latino populations engage (37–39). In the context of the present study, since not all PLWH may wish to or be able to achieve HIV viral suppression, the MI approach, which supports participants' personal decisions about HIV management without pressure or judgment, has utility.

Galarraga and colleagues carried out a substantive review of programs that used conditional economic incentives to improve HIV treatment adherence, mainly programs in clinical settings and including those grounded in behavioral economics (40). The review found that when appropriately implemented, conditional economic incentives can help PLWH improve their adherence to HIV treatment in the short-term, while incentives are in place. However, mechanisms to increase habit formation or maintain effects in the longer term warrant more investigation (40). Behavioral economics uses rewards and/or “nudges” to alter behavior and circumvent cognitive biases (41). Nudges are subtle and often indirect reminders that attempt to influence behavior through the way choices are made, taking into consideration behavioral biases. Ideally, a conditional economic incentive for behavior change (such as reaching HIV viral suppression) will align with an individual's own intrinsic motivation for behavior change, allowing the individual to build durable habits. Nudges are most effective when they are provided immediately after the desired behavior is carried out (41–44). However, it can take several months for PLWH who wish to increase HIV medication use and reach HIV viral suppression to do so (45). Thus, we use a text message and quiz question (TMQQ) component to foster engagement in the study over time and serve as a reminder about the larger goal of achieving viral suppression (described in more detail in Methods).

Financial rewards in the form of lottery prizes or fixed compensation amounts have been used in past research to reward longer-term behavior change. Prize drawings leverage the cognitive bias of overestimating small probabilities (leading individuals to participate in the prize drawing because they overestimate their chance of winning) and also increase salience (prizes keep a behavior high on a person's mental priority list) (43). On the other hand, participants in low-income contexts may actually prefer a fixed compensation amount over a lottery prize (11).

Overall, the results from our past study highlighted that the conceptual approach taken and the intervention activities in the S-CAP intervention warranted further exploration. We also identified a number of ways the intervention could be improved. For example, results indicated that the lottery prize structure was overly complicated, some participants would have preferred a fixed compensation amount for viral suppression over a lottery prize (although fixed compensation vs. a lottery prize have not yet been directly compared in the literature), participants requested additional MI counseling sessions, and the TMQQ period may have been too brief. We applied these lessons learned to the present study.

As is common in intervention research, the S-CAP intervention tested in the previous study consisted of a number of intervention components that were combined into a single “packaged” intervention. One disadvantage of testing packaged interventions in what is often called the classical approach (generally using the randomized controlled trial design) is that, if found efficacious/effective, it is not possible to determine which of the components contributed to its efficacy, if some components performed better than others, or if some components had counter-productive effects on others. Further, when packaged interventions are not found efficacious/effective in a randomized controlled trial, it is not possible to determine if any of the components showed promise, or to determine what the next steps in the program of research should be. The multiphase optimization strategy (MOST) framework solves these problems through the systematic testing of individual intervention components and their interactions using a variety of designs (46).

The MOST framework is inspired by engineering and has three stages: preparation (identifying intervention components, developing a conceptual model, and identifying the “optimization objective” to guide future decisions about whether or how to combine components), optimization (evaluating the effects of components, applying the optimization objective to create a new multi-component intervention, if appropriate), and evaluation (testing the new optimized intervention in a randomized controlled trial) (46). The present study is grounded in the MOST framework. It is a pilot optimization trial that uses mixed methods and an efficient factorial design to examine the acceptability, feasibility, and preliminary evidence of effects of three separate intervention components derived from the results of the previous S-CAP intervention study, described in more detail elsewhere (11). Because the present study is exploratory and not powered for efficacy, it aligns most closely with the MOST framework's preparation phase. The three components explored in the present study are: (1) a financial reward for viral suppression (fixed compensation vs. a lottery prize), (2) weekly TMQQs for 21 weeks, and (3) three MI counseling sessions (the components are described in more detail below.) The factorial design permits a more precise exploration of each intervention component than the classical approach to testing packaged interventions allows.

## 2. Methods

### 2.1. Overview

The present mixed methods study is a pilot optimization trial, grounded in the MOST framework. The proposed study took place between 9/2020 and 1/2022 in the New York City metropolitan area, a COVID-19 epicenter. The study was carried out entirely virtually, as in-person activities with human subjects were prohibited at our institution due to COVID-19 restrictions. The study's primary outcome was HIV viral suppression, and viral load levels were a secondary outcome. We used an efficient factorial design to explore three behavioral intervention components, each designed to address specific barriers to HIV viral suppression in this population. The goals of the present study are to examine the acceptability and feasibility of the intervention components and explore preliminary evidence of their effects on factors believed to mediate changes in the primary outcome and on the primary and secondary outcomes, in order to inform future research. We enrolled 80 African American/Black and English-speaking Latino PLWH with non-suppressed HIV viral load (>200 copies/mL). Participants received a baseline assessment and follow-up assessments at 4- and 8-months post-baseline. With support of the study team, they provided a recent laboratory report including HIV viral load levels at each of the three assessment periods. Participants were randomly assigned to one of 8 experimental conditions, each comprised of a unique combination of intervention components or component levels. Consistent with the sequential explanatory mixed methods design, we used the quantitative results to develop a set of research questions that could be addressed using qualitative data, and then results from the two analyses were integrated using the joint display method. The study used the field name “Silver Community Action Project 2” (S-CAP2). Results were interpreted in the context of the COVID-19 pandemic, which impeded HIV management and other aspects of participants' lives, and the fact that all activities were carried out virtually, almost always by phone, since participants generally did not have smartphones or computers that would allow for Telehealth. Compensation was provided to participants using the Greenphire ClinCard system, a refillable debit card for research compensation. The study was approved by the Institutional Review Board at New York University and participants gave verbal informed consent for study activities.

### 2.2. Eligibility criteria

Inclusion criteria were: 1. age 18–65 years, 2. living with HIV, 3. resides in the New York City metropolitan area, 4. can participate in research activities in English, 5. has a phone and can receive text messages, 6. has not participated in a local conditional economic incentive program for HIV viral suppression in the past month, 7. has not been enrolled in the research team's two most recent research studies (the first S-CAP study and another previous study), 8. willing to provide a recent lab report showing HIV viral load (lab test completed in the past 2 months), and 9. the lab report at screening indicates non-suppressed HIV viral load (>200 pp/mL). Although race/ethnicity were not eligibility criteria, it was anticipated that >90% of participants would be African American/Black or Latino given trends in past studies and the demographic characteristics of PLWH in New York City (>75% African American/Black or Latino; (47, 48). As shown in Table 1, all participants were either African American/Black or Latino.

**Table 1:** Sociodemographic and background characteristics and HIV-related health factors (*N* = 80).

	**Mean (SD) or %**
Age in years (M, SD)	49.0 (9.42)
Age range [min, max], in years	29.0, 62.0
**Sex, sexual orientation, and gender identity**	
Male sex assigned at birth	75.0
Female sex assigned at birth	25.0
Sexual minority (bisexual, homosexual, queer, gay, lesbian)	38.8
Transgender, gender fluid, gender identity	15.0
African American/Black (non-Latino/Hispanic)	78.8
Latino/Hispanic	16.3
High school graduate/equivalent or higher	61.3
Homeless over the lifetime	93.8
Homeless in the past year	56.3
Currently stably housed	48.8
**Indications of poverty**	
Currently employed full- or part-time	1.3
Ran out of funds for basic necessities at least monthly in the past year	58.9
Any indication of food insecurity	86.3
Receives public health insurance (e.g., Medicaid)	96.3
Receives public entitlements/assistance (e.g., food stamps, cash benefits)	100
**HIV History and HIV Health Status Indicators**	
Years living with HIV/years since HIV Diagnosis (M, SD)	20.1 (9.50)
Range of years living with HIV [min, max]	< 1, 37.0
Perinatally infected with HIV	0.0
Has taken HIV medication in the past	95.0
Years since first initiated HIV medication	17.2 (9.12)
Range of years since initiated ART [min, max]	0, 38.0
Number of HIV medication starts (range 0–288 times)	7.49 (13.2)
Longest duration of sustained HIV medication over the lifetime, in months (range 2–204 months)	36.2 (45.0)
Adherence to HIV medication—past month (range 0–100)	64.0 (37.0)
Taking any HIV medication at enrollment	81.3
If not on any HIV medication at enrollment, number of months since last dose	5.29 (5.14)
Satisfaction with HIV care (range 0–100)	77.1 (22.8)
**Substance use**	
Alcohol use at a moderate-to-high risk level	37.5
Cannabis use at a moderate-to-high risk level	51.3
Cocaine use at a moderate-to-high risk level	66.3
Polysubstance use (2+ substances excluding tobacco and alcohol) at a moderate-to-high risk level	51.3
Any substance use treatment lifetime	75.0

### 2.3. Preparation for the present study

In preparation for the present study, the research team and a Community Advisory Board created separate intervention components from the original packaged S-CAP intervention and made minor modifications to them, based on past study findings. We increased the number of MI counseling sessions from one to three, increased the length of the TMQQ period from 16 to 21 weeks, changed some specific TMs that were unclear or that participants did not find acceptable, and added a comparison between fixed compensation and a lottery prize.

### 2.4. Component levels

In this design, components have two “levels,” such as “on” (the participant receives the component) or “off” (the participant does not receive the component). In the present study, the component levels were: (A) financial rewards (fixed compensation vs. lottery prize); (B) TMQQ (on/off), and (C) MI counseling sessions (on/off). All participants also received a brief core orientation session. The core session is not evaluated, since all participants receive it.

### 2.5. Design

**Figure 1:**
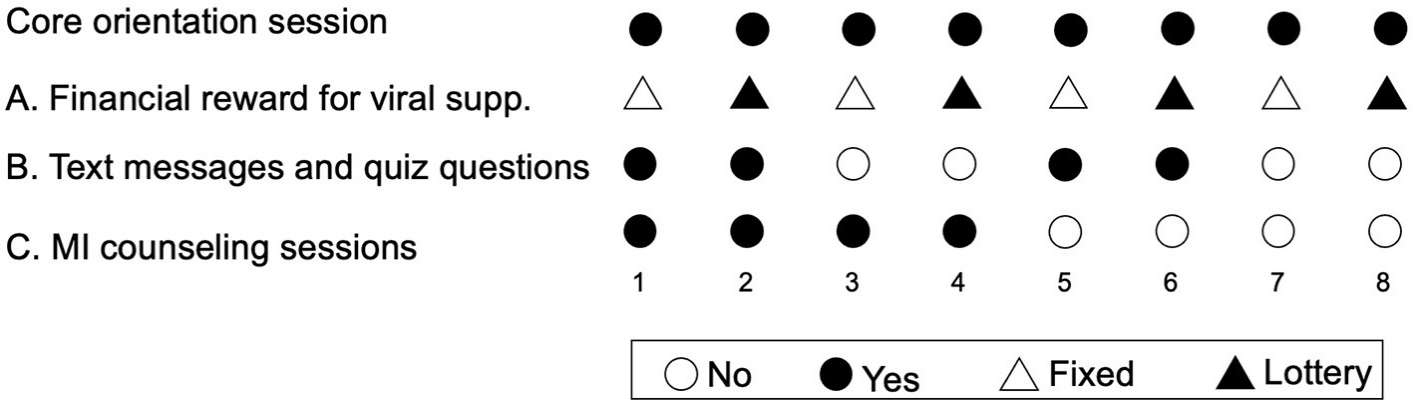
Experimental conditions in the factorial design.

A full factorial experiment with three components (also called factors), each comprising two levels, contains 2^3^ unique combinations of component levels. Thus, the factorial design comprises every possible combination of the component levels. In this case, each of the eight unique combinations of component levels constitutes a different experimental *condition*. Participant are randomly assigned to an experimental condition (Figure 1) (49). For example, in condition 1, participants receive the core session, fixed compensation for viral suppression, TMQQs, and MI counseling sessions. In condition 8, participants receive the core session and the lottery prize for viral suppression, but no other components.

### 2.6. Description of intervention components

#### 2.6.1. Core orientation session (< 60 min)

This session comprised an introduction to the study and its ethos grounded in the conceptual model described above (e.g., emphasizing support for personal decisions about HIV management and any positive change, no pressure, no judgment, and structural and cultural salience) (27, 31), a brief needs assessment, and referrals to needed services (e.g., HIV care, food pantries, housing, or clothing) to help overcome structural barriers to HIV care or medication. Participants were informed about the type of financial reward they were eligible to receive and the structure and duration of other components they were randomly assigned to receive during the core session.

#### 2.6.2. Component A: financial reward for HIV viral suppression

All participants could become eligible for a financial reward at the first follow-up (FU) assessment by achieving HIV viral suppression. The financial reward was either a fixed compensation amount or lottery-type prize. The fixed compensation amount was $300, based on the literature, input from community partners, and our previous research (11). For those randomly assigned to receive the lottery prize level, prize amounts were determined by chance. Participants had a 3/10 chance of winning $500 and a 7/10 chance of winning $250. Thus, the average lottery prize amount was $325, comparable to the fixed compensation amount. The lottery prize was determined by spinning an electronic prize wheel, similar to a roulette wheel. This was done virtually. Participants had the option of delaying receiving the financial reward until the second follow-up assessment, for example in cases where they were increasing their HIV medication adherence, but had not yet achieved viral suppression. Those who did not achieve HIV viral suppression in the study period or who were unable to provide a lab report with their HIV viral load results received a $50 participation bonus. The primary mediator for the financial reward component was motivation for HIV viral suppression.

#### 2.6.3. Component B: TMQQs

Those randomized to an experimental condition that included the “on” level of this component received TMQQs over a 21-week period (a reasonable length of time during which to engage in care, re-initiate HIV medication, change adherence patterns, and achieve HIV viral suppression, if so desired). Each week a TM was sent to participants' phones, containing health and general HIV-related information including a hyperlink to more information, as appropriate, or a motivational message (see Supplementary Table 1). Two days later, a second TM was sent with a true/false QQ based on the first TM. The QQs were not intended to be difficult but instead were designed to keep participants engaged in the study and serve as a reminder that the ultimate goal of the study was to support HIV viral suppression. The TMQQ component was implemented by the Telerivet program, which sent TMs and QQs automatically. After participants responded to the QQ, Telerivet sent additional TMs indicating whether the response was correct or not. Participants received five points for answering the true/false QQ correctly and two points if they answered, but incorrectly. They were informed they would receive $1 per point earned. The maximum compensation amount was $105 if participants answered every QQ correctly. The primary mediator for the TMQQ component was motivation, and engagement in the study, assessed as the proportion of QQs answered, to foster retention, was a secondary mediator and mechanism of action.

#### 2.6.4. Component C: MI counseling sessions (30–40 minutes each)

The component focused on eliciting participants' decisions regarding reaching HIV viral suppression and how that might be achieved if participants wished to do so. The three sessions were guided by manuals that were developed by the research team and a Community Advisory Board for the present study. The written manuals note the overall goal for the component, techniques recommended as part of MI (e.g., developing discrepancy, readiness ruler, encouraging change talk), and guidance on training on habit formation. Then, each session was guided by a sequence of activities, with sample language provided as a guide. Further, interventionists were instructed to think of the component as flexible and individualized in order to meet participants' needs and to use the manuals in that context. Session one guided participants to identify HIV-related goals, barriers, and facilitators of goals, and introduced participants to the idea of habits for HIV medication. Session two took place ~2 weeks later and reviewed goals, habits, successes, and barriers, and discussed the perceived value of sustained HIV medication adherence at levels sufficient to achieve viral suppression. The final session took place ~2 weeks after that and focused on gains made and sustaining achievements after the financial reward was received. The primary mediator for MI counseling sessions was motivation for HIV viral suppression. (Manuals are available from the corresponding author.).

### 2.7. Recruitment

Participants were recruited using a hybrid method that included advertisements placed in the medical research section of a local free newspaper, flyers posted in local community-based organizations, and peer-to-peer recruitment, where participants were compensated $15 for referring peers to the study. Peer recruiters were also able to receive a bonus of $15 in compensation per peer who provided a laboratory report to the study, as a means of using peer influence, support, and reminders to encourage the timely provision of such laboratory reports, which were challenging for participants to obtain. Most participants were recruited from peer referral 72.5% (58/80), and a minority from newspaper advertisements (25.0%, [20/80]), and through flyers posted in community-based organizations or other means (2.5%, [2/80]).

### 2.8. Study procedures)

**Figure 2:**
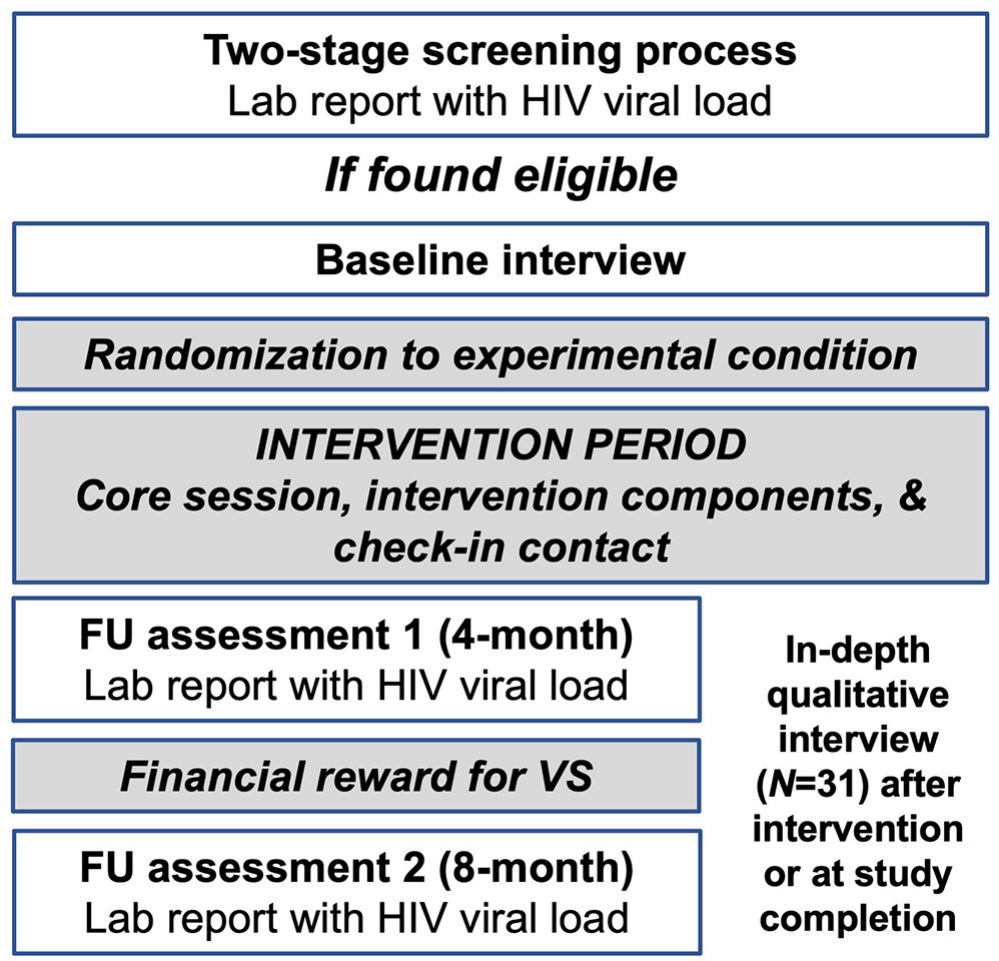
Sequence of study activities.

The study was managed in the Research Electronic Data Capture (REDCap) platform. REDCap is a cloud-based platform for data capture designed for clinical research (50, 51). Assessment batteries were programmed in REDCap and administered to participants by trained interviewers. The sequence of study activities have been outline in Figure 2.

#### 2.8.1. First screening interview

Screening for study eligibility took place in two stages. For the first screening interview, potential participants contacted the study directly by phone. Because all activities were virtual, we obtained verbal informed consent following an IRB-approved script, and then study staff led participants through a brief structured assessment in the computer-assisted personal interview (CAPI) format on the REDCap platform that assessed eligibility criteria. Gender identity, sex assigned at birth, and race/ethnicity were assessed but were not eligibility criteria.

#### 2.8.2. Second screening interview

Those found preliminarily eligible at this stage were told they might be eligible for the research study, pending confirmation of non-suppressed HIV viral load on a recent laboratory report (HIV viral load assessed in the past 2 months). If potential participants were interested, the research staff discussed strategies participants could use to obtain new or existing laboratory reports without cost. Participants were asked to provide the laboratory report in an electronic format prior to the second screening interview (e.g., a photograph or screenshot sent electronically to a password-protected computer) or have their healthcare facility provide the report to the study electronically. Electronic faxes from health care settings were received by a computer-based application on a password-protected computer. Challenges to obtaining laboratory reports were common and included participants not recently attending HIV care visits and therefore not having a recent lab report, their having inconsistent access to cell phones or cell phone service being cut off, not being certain how to request or not feeling comfortable requesting a lab report from the provider, or not knowing how to create a screenshot or take a photograph to send lab results to the study electronically. These barriers were overcome by walking participants through the process of obtaining records, helping them take a problem-solving approach to barriers, and offering to contact the provider directly (with participants' signed consent). Participants typically required assistance obtaining the laboratory report (>90% of the time).

During the second screening interview, HIV viral load values were entered into REDCap and we then determined study eligibility based on HIV viral suppression status. Lab reports were scanned as needed and the electronic version was loaded into REDCap. No paper copies were retained, nor were electronic copies of records stored on computer hard drives, to protect participant confidentiality. Because participants generally requested their own records from providers or provided their records to the study, they were not required by the study to sign a Health Insurance Portability and Accountability Act (HIPAA) consent form. However, participants did sign a HIPAA form in cases where we were asked to contact the providers directly (we contacted providers in ~10% of cases). Participants received $30 for providing the laboratory report to the study and $10 for the second screening interview.

#### 2.8.3. Enrollment and baseline assessment

Those found eligible provided verbal informed consent and completed a structured baseline assessment battery in the CAPI format on the REDCap platform lasting ~60 min. Participants received $30 for the baseline assessment.

#### 2.8.4. Randomization to an experimental condition

After completing the baseline assessment, participants were randomly assigned to one of the eight different experimental conditions, using a randomization table created by the study's biostatistician and programmed in REDCap. Regarding the order in which components were delivered, the core session was provided first. MI sessions were provided next for those assigned to receive that component, followed by the TMQQs for those assigned to receive it. Financial rewards were provided last for all participants.

#### 2.8.5. Check-in contact

At ~8 weeks post-baseline, participants engaged in a check-in call (< 30 min). The goal of the check-in contact was to identify and solve any problems preventing engagement in the study. Participants received $25 for the check-in contact along with any TMQQ compensation earned thus far, for participants assigned to the TMQQ component.

#### 2.8.6. First FU assessment (~4-months post-baseline)

Prior to the first FU assessment, participants were contacted and asked to provide a recent lab report with HIV viral load levels (regardless of whether they were HIV virally suppressed or not). When laboratory reports were obtained, the FU assessment was scheduled and carried out in CAPI in the REDCap platform (lasting ~60 min). Participants received $40 for providing the laboratory report and $30 for the FU assessment.

#### 2.8.7. Determination of financial reward

Participants who were assigned to the fixed compensation category received the appropriate compensation based on whether they achieved HIV viral suppression. Participants assigned to the lottery prize were able to hear or watch (if on a Voice over Internet protocol) study staff spin a virtual prize wheel for them and earned the lottery prize based on whether they achieved HIV viral suppression, and chance. Otherwise, the participation bonus was provided.

#### 2.8.8. Second FU assessment (~8-months post-baseline)

Procedures for the second FU assessment were similar to the first: participants were contacted in advance to obtain the laboratory report, and then the FU assessment was scheduled and carried out. Participants received $40 for providing the laboratory report and $30 for the second FU assessment.

### 2.9. Procedures for qualitative interviews

Participants were recruited for in-depth qualitative interviews at one of two-time points. The first in-depth qualitative interview was conducted after the first FU assessment and the second in-depth interview took place after the second FU interview. Those who engaged in the first in-depth interview were not recruited for the second interview. Interviews lasted between 60 and 90 min, and were conducted virtually. Trained interviewers used a semi-structured interview guide as a template for the interview. Interviews were audio-recorded and professionally transcribed. Participants were compensated $30 for participating in an interview. For both interviews, participants were purposively sampled for maximum variability on key indices including sex, whether they achieved HIV viral suppression, time living with HIV, and experimental condition assigned to. A total of 16 participants were interviewed after the first FU and a total of 15 participants were interviewed after the second FU (total qualitative sample size was 31). Participants were compensated $30 for the in-depth interview.

### 2.10. Quantitative measures

#### 2.10.1. Sociodemographic and background characteristics

Structured instruments developed specifically for HIV-affected populations in high-risk contexts such as the population under study here were used to assess relevant quantitative domains, including age, sex assigned at birth, gender identity, sexual minority status (identifies as gay, lesbian, bisexual, queer, or other non-heterosexual), race/ethnicity, education level (high school graduate or equivalent or higher), history of homelessness (homeless over the lifetime, homeless in the past year), and whether currently stably housed (that is, the residence is not temporary [such as a single-room occupancy hotel] or a location unfit for human habitation, including living on the streets). We assessed indications of poverty such as frequency of running out of funds for basic necessities at least monthly in the past year, any indication of food insecurity on a three-item scale, any receipt of public benefits such as food stamps or cash assistance, whether receives public health insurance (e.g., Medicaid), and whether currently employed full- or part-time (52). We assessed a range of HIV indices using a version of the HIV Cost and Services Utilization Study instrument (HCSUS) (53) including: years since first HIV diagnosis, whether perinatally infected with HIV, whether has taken HIV antiretroviral therapy in the past, years since first initiated HIV antiretroviral therapy, number times has stopped and started HIV antiretroviral therapy (a numerical response), the longest duration of sustained HIV antiretroviral therapy use in months (a numerical response), adherence to HIV antiretroviral therapy doses over the past 4 weeks on a visual analog scale (VAS; range 0–100% of prescribed doses taken), if not on HIV antiretroviral therapy at enrollment, number of months since last dose (a numerical response), and satisfaction with HIV care (range 0–100). Patterns of substance use were assessed using the World Health Organization Alcohol, Smoking, and Substance Involvement Screening Test (WHO ASSIST) which provides scoring algorithms to distinguish substance use at moderate-to-high risk vs. low-risk levels (54). We assessed engagement in any substance use treatment in the past (e.g., outpatient drug treatment, detox, inpatient drug treatment, methadone maintenance treatment program, 12-step or self-help meetings like Alcoholics Anonymous [AA] or Narcotics Anonymous [NA]), an indicator of past concerns about substance use (recoded as yes if any substance use treatment was reported). Physical and mental health were assessed using the SF-12 measure, a self-reported outcome measure assessing the impact of health on an individual's everyday life (55). We created T-scores from the SF-12 items; namely, weighted linear composite scores using weights presented by Ware and colleagues (55). The normative mean for composite scores in the 1995 general U.S. population was 50. In addition to physical and mental health composite scores, we also used the SF-12 items to create the SF-6D preference-based measure of health described by Brazier and Roberts (2004) (56). We used the SF-12 items (57) to create the SF-6D preference-based measure of health described by Brazier and Roberts (2004) (58). SF-6D scores can range from 0.35 to 1.0 with higher values indicating better health. The recent median SF-6D score for the adult United States population is 0.8 (59).

#### 2.10.2. Motivation

We assessed motivation for (1) HIV care attendance and motivation to (2) take HIV antiretroviral therapy (if taking HIV antiretroviral therapy at all at the time of enrollment) or increase HIV antiretroviral therapy adherence (if taking HIV antiretroviral therapy but not at a sufficient level to achieve viral suppression), and motivation for (3) undetectable viral load. Based on past research, motivation was conceptualized as how important a behavior or outcome is to an individual and how confident they are they can engage in the behavior or achieve the outcome (60). Importance of the behavior was rated on a 1–10 scale (e.g., On a scale of 1–10, how important is it to you today to significantly increase how often you take HIV medication, where 1 is not important at all, and 10 is extremely important?), followed by the participant's confidence that they could engage in the behavior (e.g., On a scale of 1–10, how confident are you that you could significantly increase how often you take HIV medication, where 1 is not at all confident and 10 is extremely confident?). Thus, “motivation” for a behavior was operationalized as the mean of the importance score and the mean of the confidence score and ranged from 1 to 10; higher values indicated higher motivation for the behavior (60).

#### 2.10.3. HIV treatment engagement

We assessed HIV treatment engagement by (1) assessing HIV medication adherence (range 0–100) and we assessed (2) the amount of HIV medication taken in the past 4 weeks using a visual analog scale ranging from 0 to 100% (53).

#### 2.10.4. Acceptability

A version of the Client Satisfaction Survey (61) was adapted to the present study by the research team and reviewed by the community advisory board for comprehensiveness and clarity. The revised Client Satisfaction Survey was used to assess the acceptability of the study overall and of aspects of the intervention components. A total of 19 items were assessed such as “the S-CAP2 staff understand the treatment needs of people of my racial, ethnic, or cultural group,” and “the chance to win a financial reward as part of the S-CAP2 study played a role in my recent HIV medication decisions.” Items were rated on two types of Likert scales depending on the item (poor, fair, good, very good, excellent, rarely or never, sometimes, most times, and all of the time) and coded to reflect the proportion who endorsed the item as “very good to excellent” or “most times to all of the time.” An activity was considered acceptable if 70% or more of participants endorsed it as “very good to excellent” or “most times to all of the time.” Some questions were asked at the second follow-up assessment only.

#### 2.10.5. Feasibility

Study feasibility was defined as the proportion of participants attending assigned components. The study or a component was considered feasible if 70% or more of the participants engaged in the activity.

#### 2.10.6. Primary and secondary outcomes

HIV viral suppression (<200 copies/mL) and log_10_ HIV viral load level were assessed by laboratory reports provided by the participant's HIV primary care site.

#### 2.10.7. Confidence intervals

We calculated 95% confidence intervals (CIs) for the main effect of each intervention component in a model for log_10_ HIV viral load at the second FU. Missing data were imputed 80 times with a chained equation approach where baseline HIV viral load, first FU HIV viral load, and all intervention components were included in the imputation model.

### 2.11. Qualitative interview guides

We used a semi-structured guide developed by the research team, which included experts on African American/Black and Latino PLWH, sexual and gender minorities, behavioral economics, and the HIV care continuum. The interview guide was structured as a series of suggested questions and prompts. The guide directed the interviewer from general to more specific questions in each of the following sections and was divided in two parts. Part 1 of the interviews focused mainly on participants' views on the intervention components: (1) general overview of the participant's experience in the project (e.g., What stands out to you most about the S-CAP2 project?); (2) experiences with fixed compensation or prize (e.g., Was the idea of receiving [fixed compensation or lottery prize] based on undetectable viral load fair? Motivating? Interesting? Confusing? Did the [compensation/prize] make you feel pressured in any way?); (3) views on sustaining viral load, where relevant (e.g., Do you plan to continue taking HIV medication and/or sustain an undetectable viral load after the [compensation/prize]? Why or why not? (4) experiences with the TMQQ component, for those assigned to receive it (e.g., What do you think about the text messages you have received? Are they easy to read? Hard to read? Too long? Too short? Are they helpful in any way? If so, how? Are they unhelpful in any way?); (5) the TMQQ component's potential effects on HIV decisions and behavior (e.g., Did the TMQQs have any influence or effect on your decision to take HIV medication or not? Why or why not?); (6) experiences with MI counseling sessions for those assigned to receive that component (e.g., Were the sessions helpful for you? Were you able to create habits around your HIV medication?); (7) effects of MI counseling sessions on HIV decisions and behavior (e.g., Did the sessions play a role in your decisions to take HIV medication or create any other health goals? Why or why not?).

Part 2 of the interview guides focused on the context of HIV management and experiences in the S-CAP2 study more generally. Questions included: (1) acceptability, feasibility, and safety (e.g., Has there been anything about S-CAP2 that you think has been particularly unhelpful? Helpful? What do you think should be included that was not included?); (2) the experience of virtual intervention (e.g., S-CAP2 was conducted on the phone because of COVID, was that OK for you? Do you feel you had a connection to or relationship with S-CAP2 even though you never met the team in person?); (3) lab reports (e.g., How was the process of getting your lab reports? What got in the way of getting your lab reports? Was the S-CAP2 project helpful with respect to getting lab reports?); and (4) COVID-19 (e.g., Looking back, in what ways did the COVID pandemic influence your HIV management? Did you get tested for COVID? Have you been vaccinated?).

### 2.12. Quantitative data analysis

Descriptive statistics were presented by the time of assessment (baseline, FU1, and FU2) and by the levels of each intervention component, with percentages for categorical variables and means and standard deviations for continuous variables. Following recommendations from NIH (62) and in the methods literature (63), we did not perform null-hypothesis significance testing with these pilot data. All analyses were conducted with the R statistical computing environment (64).

### 2.13. Qualitative data analyses

Analyses of qualitative data followed a directed content analysis approach that was both inductive and theory-driven (65). Analyses were carried out in the Dedoose platform. We started with an initial list of “start codes” and their operational definitions that was generated by the primary qualitative analyst, who is a medical anthropologist. This initial start code list was informed by the theories and perspectives framing the study. Codes were generated that reflected structural barriers (e.g., quality of housing, poverty), culture and race/ethnicity (e.g., experiences of discrimination, medical distrust); and substance use management; autonomy, competence, relatedness, and other factors known to promote or impede engagement along the HIV care continuum (e.g., mental health distress). Using this scheme, the primary analyst coded interview transcripts along with an additional trained qualitative researcher. During the coding process, codes were refined, clarified, and/or broadened; for example, when new codes were identified. Discrepancies in codes and coding between the data analysts were resolved by consensus. Then, the interview transcripts were recoded using the final coding frame. Further, a subset of transcripts were coded using the final coding frame by three other members of the research team. Codes were then combined into larger themes and sub-themes in an iterative process led by the two main data analysts and in collaboration with an interpretive community of research team members, which included people who identify as cisgender men and women, people who are transgender, gender non-binary, or gender-fluid, people from White, African American/Black, Asian, and Latino backgrounds, and PLWH (66, 67). Methodological rigor of the analysis was monitored continually in several ways. An audit trail of process and analytic memos was maintained (68). Analysts engaged in debriefing sessions approximately monthly with the interpretive community. The primary analysts and the interpretive community attended to the potential effects of the team's positionality related to power and privilege, sex, gender, race/ethnicity, health, and socioeconomic status throughout the data collection process through reflection and training that focused on how these factors might affect interviewing and data analytic processes (69, 70).

### 2.14. Data integration procedures

Data integration followed procedures outlined by Fetters and colleagues (71) and used the joint display method (71). A joint display is a state-of-the-art visual tool (i.e., a side-by-side visual presentation of results) to integrate data sources. The process brings about new insights beyond the information gained from the separate quantitative and qualitative results. Data integration was carried out by the interpretive community made up of research team members in an iterative process in which each joint display table revealed insights about the merged findings that shaped subsequent iterations. Thus, joint displays are both a method and a cognitive framework for data integration and facilitate the production of new inferences (71). Beginning with the major quantitative findings, the interpretive community assessed areas of convergence and divergence between the quantitative results and the primary themes in the qualitative data. To do so, we used an informational matrix to compare results at a granular level (finding-by-finding) (71). Then, we explored primary qualitative findings that may not be present in the quantitative results. The results from this data integration effort were summarized and presented in a joint display table.

## 3. Results

### 3.1. Demographics and other characteristics at baseline

We recruited 80 participants who had a mean age of 49 years, were 75% cisgender men, 15% transgender, 79% non-Hispanic African-American/Black, and 39% sexual minority (Table 1). Approximately 61% had completed high school or equivalent secondary education. Almost none were employed (1%) and the majority (59%) ran out of funds for basic necessities at least monthly in the past year. Participants had been diagnosed with HIV for an average of 20 years. Most (81%) reported taking HIV medication at baseline, but self-reported adherence on the 0–100 visual analog scale was modest (mean = 64). About half (51%) had use of two or more substances other than tobacco or alcohol at moderate-to-high risk levels, and most (75%) had been in substance use treatment in the past. Other sociodemographic and background characteristics are presented in Table 1.

### 3.2. Enrollment and feasibility

**Figure 3:**
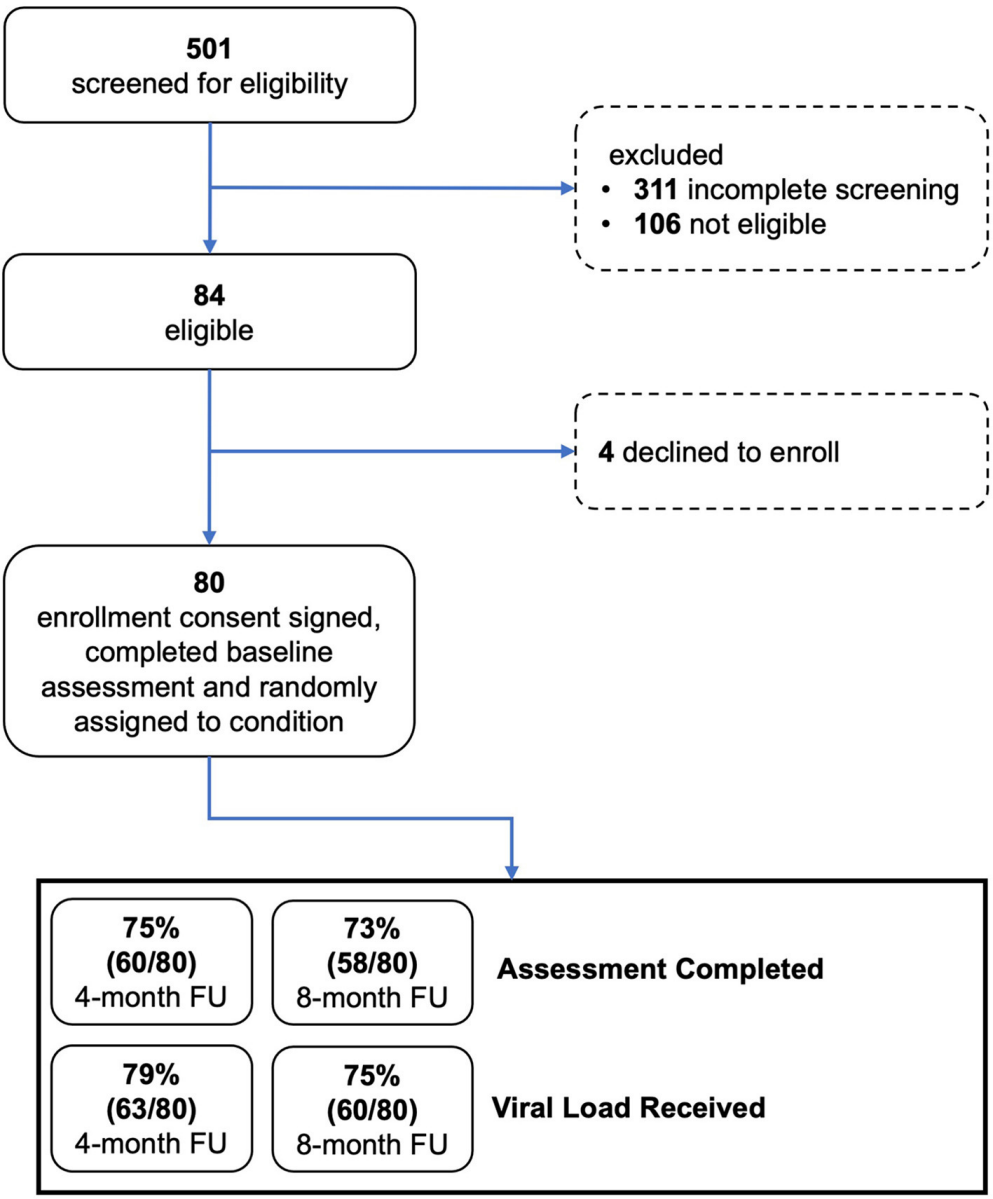
CONSORT diagram.

Figure 3 provides data on study screening, enrollment, and participation in study activities, consistent with the Consolidated Standards of Reporting Trials (CONSORT) model. Among potential participants who completed the two-stage screening process, 84 were eligible and 80 (95% of eligible) went on to enroll and were randomly assigned to an experimental condition. Approximately 75% of participants completed an assessment and viral load testing at the 4- and 8-month FU assessments. As shown in Table 2, most enrolled participants (96%) completed the core intervention session. Most participants assigned to MI sessions completed all three of those sessions (38 of 40, 95%). Most participants assigned to the TMQQ component (32 of 39, 82%) answered at least one QQ, on average responding to 11 of the 21 QQs.

**Table 2:** Feasibility of study activities.

	**N (%) or M (SD)**
Completed core intervention session	78/80 (97.5)
Completed all three MI counseling sessions	38/40 (95.0)
Check-in contact completed	74/80 (92.5)
**Text messages (TM) and quiz questions (QQ)**	
Answered at least one QQ	32/39 (82.1)
Number QQs answered (of 21 max.) [Mean, SD]	*10.7 (8.0)*

### 3.3. Acceptability and self-reported influence of S-CAP2 as a whole and intervention components

Table 3 shows responses to items assessing acceptability of the overall project as well as specific intervention components. Overall, most participants rated the activities and services of the project as very good or excellent at the 4-month (82%) and 8-month (69%) FUs. Participants also rated the information received as helpful or very helpful. Being treated like an individual, respect for privacy, and understanding of the needs of racial, ethnic, or cultural groups were all rated favorably as well. Most participants indicated the project increased their desire to take HIV medication, and planned to continue taking HIV medication after the end of the project.

**Table 3:** Intervention acceptability and participant perspectives (*N* = 80).

**Acceptability**	**FU1 (*N* = 60)**	**FU2 (*N* = 58)**
Overall, I think that the activities and services in the S-CAP2 project are Very Good/Excellent	49 (81.7%)	40 (69.0%)
The information I have received in the S-CAP2 project has been Helpful/Very Helpful	59 (98.3%)	58 (100%)
The S-CAP2 staff here treat me like I am an individual with unique needs and concerns	51 (85.0%)	51 (87.9%)
The S-CAP2 staff respect my privacy All of the Time	58 (96.7%)	57 (98.3%)
The S-CAP2 staff here understand the treatment needs of people of my racial, ethnic, or cultural group	57 (95.0%)	56 (96.6%)
**Participant perspectives on the study and components**		
The S-CAP2 project increased my desire to take HIV medication	52 (86.7%)	46 (79.3%)
Do you think you will continue to take HIV medication after the S-CAP2 project ends? (yes)	57 (95.0%)	58 (100%)
**Lottery prize**	**FU1 (*****N*** = **29)**	**FU2 (*****N*** = **30)**
The chance to win a prize for achieving undetectable viral load as part of the S-CAP2 project played a role in my recent HIV medication decisions	15 (51.7%)	15 (50.0%)
Because of the chance to win a prize as part of the S-CAP2 project, I tried to achieve HIV undetectable viral load	18 (62.1%)	13 (43.3%)
**Fixed compensation**	**FU1 (*****N*** = **31)**	**FU2 (*****N*** = **28)**
The chance to receive compensation for achieving undetectable viral load in the S-CAP2 project played a role in my recent HIV medication decisions	15 (48.4%)	13 (46.4%)
Because of the chance to receive compensation in the S-CAP2 project, I tried to achieve HIV undetectable viral load	13 (41.9%)	11 (39.3%)
**Text messages and quiz questions (TMQQ)**	**FU1 (*****N*** = **32)**	**FU2 (*****N*** = **27)**
Overall I think that the text messages I received as part of the S-CAP2 project are Very Good/Excellent	21 (65.6%)	17 (63.0%)
Receiving TMQQs to earn points as part of the S-CAP2 project played a role in my recent HIV medication decisions	22 (68.8%)	14 (51.9%)
Because of the TMQQs, I took HIV medication more often than I did in the past	19 (59.4%)	11 (40.7%)
**MI counseling sessions**	**FU1 (*****N*** = **30)**	**FU2 (*****N*** = **25)**
Meeting with the S-CAP2 staff to discuss my goals and learn about habits as part of the S-CAP2 project played a role in my recent HIV medication decisions	22 (73.3%)	19 (76.0%)
Because of meeting with the S-CAP2 staff to discuss my goals and habits, I took HIV medication more often than I did in the past	25 (83.3%)	21 (84.0%)
Because of the meetings with the S-CAP2 staff, I tried to achieve HIV undetectable viral load	26 (86.7%)	20 (80.0%)

TMQQs were designed in part to support engagement in the study. Among participants assigned to the TMQQ component, more than half found the messages very good or excellent at the 4-month (66%) and 8-month (63%) FUs. More than half of participants assigned to this component said the TMQQs played a role in HIV medication decisions and led them to take more medication at the 4-month FU, but ratings of the importance of TMQQs seemed to decrease at the second, 8-month FU.

Regardless of whether a participant was assigned to the fixed compensation or lottery prize, about half said the financial reward played a role in decisions about HIV medication or led them to try achieving undetectable viral load. Nearly all participants said they planned to continue taking HIV medication after receiving the financial reward at the end of the project.

Most participants assigned to the MI counseling sessions component said the sessions played a role in their HIV medication decisions and led them to take more medication at both FUs. Most participants assigned to this component also said the MI counseling sessions led them to try achieving undetectable viral load.

### 3.4. Motivation

Regardless of assignment to components, ratings of motivation for taking HIV medication, attending HIV care, and having an undetectable viral load were high at baseline and increased from baseline to FU (Tables 4a–c). Variability in ratings of motivation also generally decreased as more participants approached or reached the ceiling of the 0–100 scale in follow-ups.

**Table 4a:** Motivation, health-related quality of life, HIV treatment engagement, and HIV viral load over time (Mean [SD] or percent).

	**Baseline**		**Follow-Up One**		**Follow-Up Two**	
	**Fixed (*****n*** = **41)**	**Lottery (*****n*** = **39)**	**Fixed (*****n*** = **31)**	**Lottery (*****n*** = **29)**	**Fixed (*****n*** = **28)**	**Lottery (*****n*** = **30)**
**Motivation (0–100)**						
Motivation for HIV Care	88.7 (19.0)	92.4 (13.1)	95.0 (8.37)	95.4 (6.21)	94.3 (10.2)	97.2 (4.63)
Motivation for High HIV Medication Adherence	81.5 (24.7)	83.7 (21.0)	91.3 (9.97)	95.7 (7.53)	94.7 (11.1)	95.9 (5.80)
Motivation for Undetectable Viral Load	86.8 (22.1)	91.3 (13.5)	90.4 (18.5)	92.8 (9.52)	92.1 (12.7)	93.0 (12.7)
**SF-12 health-related quality of life**						
SF-6D Health Utility score	0.705 (0.188)	0.684 (0.187)	0.742 (0.188)	0.742 (0.197)	0.761 (0.207)	0.698 (0.174)
SF-12 Physical Health T-score	44.4 (12.2)	43.4 (10.8)	46.6 (11.4)	43.8 (10.5)	47.5 (11.0)	45.1 (11.4)
SF-12 Mental Health T-score	46.5 (12.7)	44.9 (12.2)	48.2 (10.9)	49.6 (10.2)	47.0 (10.3)	44.7 (11.7)
**HIV treatment engagement**						
HIV Medication Adherence (0–100)	66.1 (38.3)	50.3 (40.1)	65.3 (28.3)	72.8 (26.3)	67.9 (24.9)	79.8 (21.0)
HIV Medication Taken in Past 4 Weeks	34 (82.9%)	28 (71.8%)	30 (96.8%)	27 (93.1%)	28 (100%)	30 (100%)
**HIV viral load**						
log_10_ HIV viral load^†^	4.06 (0.90)	3.78 (0.91)	3.40 (1.61)	3.26 (1.34)	3.44 (1.64)	2.85 (1.44)
Suppressed (viral load < 200)	0 (0%)	0 (0%)	9 (29%)	7 (22.6%)	7 (24.1%)	13 (41.9%)

^†^At Follow-Up One, viral load was available for 31 participants assigned to fixed compensation and 31 participants assigned to lottery prize. At Follow-Up Two, viral load was available for 29 participants assigned to fixed compensation and 31 participants assigned to lottery prize.

Available for 29 participants assigned to fixed compensation and 31 participants assigned to lottery prize.

**Table 4b:** Motivation, health-related quality of life, HIV treatment engagement, and HIV viral load over time (Mean [SD] or percent).

	**Baseline**		**Follow-Up One**		**Follow-Up Two**	
	**No TMQQ (*****n*** = **41)**	**Yes TMQQ (*****n*** = **39)**	**No TMQQ (*****n*** = **28)**	**Yes TMQQ (*****n*** = **32)**	**No TMQQ (*****n*** = **31)**	**Yes TMQQ (*****n*** = **27)**
**Motivation (0–100)**						
Motivation for HIV Care	89.1 (19.7)	92.0 (12.1)	96.0 (6.05)	94.5 (8.35)	95.6 (6.83)	95.9 (9.06)
Motivation for High HIV Medication Adherence	82.0 (26.0)	83.2 (19.3)	93.9 (8.95)	92.8 (9.35)	96.0 (6.03)	94.5 (11.2)
Motivation for Undetectable Viral Load	89.2 (19.8)	88.7 (17.1)	91.8 (17.0)	91.4 (12.8)	90.3 (15.4)	95.2 (7.86)
**SF-12 health-related quality of life**						
SF-6D health utility score	0.671 (0.188)	0.719 (0.184)	0.682 (0.196)	0.794 (0.172)	0.729 (0.203)	0.728 (0.182)
SF-12 Physical health T-score	42.2 (12.3)	45.8 (10.4)	42.8 (11.5)	47.3 (10.2)	45.8 (11.8)	46.8 (10.5)
SF-12 Mental health T-score	44.4 (14.0)	47.1 (10.6)	45.7 (12.3)	51.7 (7.77)	46.1 (12.0)	45.5 (10.1)
**HIV treatment engagement**						
HIV Medication adherence (0–100)	62.0 (40.8)	54.6 (38.8)	71.3 (25.7)	66.8 (29.0)	74.6 (23.4)	73.4 (24.1)
HIV Medication taken in Past 4 Weeks	33 (80.5%)	29 (74.4%)	27 (96.4%)	30 (93.8%)	31 (100%)	27 (100%)
**HIV viral load**						
log_10_ HIV viral load^†^	4.01 (0.87)	3.83 (0.96)	3.35 (1.45)	3.30 (1.51)	2.84 (1.45)	3.45 (1.63)
Suppressed (viral load < 200)	0 (0%)	0 (0%)	8 (25.8%)	8 (25.8%)	11 (35.5%)	9 (31.0%)

^†^At Follow-Up One, viral load was available for 31 participants assigned to TMQQ and 31 participants not assigned to TMQQ. At Follow-Up Two, viral load was available for 29 participants assigned to TMQQ and 31 participants not assigned to TMQQ.

Assigned to TMQQ and 31 participants not assigned to TMQQ.

**Table 4c:** Motivation, health-related quality of life, HIV treatment engagement, and HIV viral load over time (Mean [SD] or percent).

	**Baseline**		**Follow-Up One**		**Follow-Up Two**	
	**No MI (*****n*** = **40)**	**Yes MI (*****n*** = **40)**	**No MI (*****n*** = **30)**	**Yes MI (*****n*** = **30)**	**No MI (*****n*** = **33)**	**Yes MI (*****n*** = **25)**
**Motivation (0–100)**						
Motivation for HIV Care	95.6 (6.25)	85.4 (21.3)	95.3 (6.69)	95.1 (8.06)	96.0 (6.49)	95.4 (9.53)
Motivation for High HIV Medication Adherence	89.1 (16.5)	76.1 (26.5)	91.8 (9.21)	95.1 (8.82)	95.9 (5.67)	94.5 (11.6)
Motivation for Undetectable Viral Load	93.0 (13.1)	84.9 (22.0)	91.6 (9.45)	91.5 (18.8)	91.5 (14.5)	94.0 (9.63)
**SF-12 health-related quality of life**						
SF-6D Health utility score	0.704 (0.188)	0.685 (0.187)	0.759 (0.183)	0.725 (0.199)	0.761 (0.170)	0.685 (0.212)
SF-12 Physical Health T-score	45.2 (10.6)	42.7 (12.3)	46.7 (11.3)	43.7 (10.6)	48.4 (9.98)	43.4 (12.1)
SF-12 Mental Health T-score	45.3 (11.9)	46.1 (13.1)	48.7 (9.35)	49.1 (11.7)	46.2 (10.2)	45.3 (12.2)
**HIV treatment engagement**						
HIV medication adherence (0–100)	61.4 (41.2)	55.4 (38.6)	70.1 (24.1)	67.7 (30.7)	73.2 (26.3)	75.2 (19.7)
HIV medication taken in past 4 weeks	31 (77.5%)	31 (77.5%)	28 (93.3%)	29 (96.7%)	33 (100%)	25 (100%)
**HIV viral load**						
log_10_ HIV viral load^†^	3.80 (0.87)	4.04 (0.94)	3.26 (1.38)	3.41 (1.59)	2.96 (1.43)	3.36 (1.71)
Suppressed (viral load < 200)	0 (0%)	0 (0%)	8 (24.2%)	8 (27.6%)	12 (35.3%)	8 (30.8%)

^†^At Follow-Up One, viral load was available for 29 participants assigned to MI sessions and 33 participants not assigned to MI sessions. At Follow-Up Two, viral load was available for 34 participants assigned to MI sessions and 26 participants not assigned to MI sessions.

### 3.5. Health-related quality of life

Health utility, physical health, and mental health scores on the SF-12 are presented in Tables 4a–c. Physical and mental health T-scores indicate participants' health was lower than for the average adult (normative mean = 50). Health was generally stable over time and similar regardless of intervention components assigned, with any differences between groups or time points small relative to the standard deviations of each health variable.

### 3.6. HIV treatment engagement and HIV medication adherence

The percentage of participants taking any medication in the past 4 weeks was high at baseline and increased in FUs, regardless of intervention components assigned (Tables 4a–c). Self-reported HIV medication adherence was ~64 on the 0–100 scale at baseline. Participants assigned to the lottery prize started with lower adherence (50 vs. 66%) and increased more than participants assigned to fixed compensation. At the second FU, adherence was 80 (sd = 21) among those assigned to the lottery prize and 68 (sd = 25) among those assigned to receive fixed compensation. At each FU point, medication adherence was similar regardless of assignment to the TMQQ and MI counseling sessions components.

### 3.7. HIV Viral Load by laboratory report

HIV viral load decreased from baseline to FU and the percentage of participants with suppressed viral load increased from baseline to FU (Tables 4a–c). Overall, log_10_ viral load was ~4.0 at baseline (sd ≈ 0.9). A total of 39.4% (26/66) of those who provided laboratory reports at FU evidenced viral suppression (32.5% [26/80] among the full sample; data not shown in Table 4).

At the second FU, participants assigned to the lottery prize had a viral load about 0.6 log_10_ lower than participants assigned to receive fixed compensation. At the second follow-up, participants assigned to the lottery prize were more likely to have viral suppression (42%) than those assigned to the receive fixed compensation (24%). When considering TMQQ and MI counseling sessions components, about one-third of participants had viral suppression at FUs and log_10_ viral load was substantially reduced relative to baseline (~0.75 log_10_ reduction) regardless of whether these components were assigned or not. We also examined the relationship between the TMQQ component and participation in post-baseline activities: check-in contact, FU1, and FU2. A total of 64% of those assigned to receive the TMQQ component engaged in all three of these activities compared to 61% of those who were not assigned to receive the TMQQ component (data not shown in Table 4).

As noted above, we calculated 95% CIs for the main effect of each intervention component in a model for log_10_ viral load at the second FU (data not shown in Table 4). In the pooled analysis model with baseline viral load as a covariate, all confidence intervals for intervention component main effects included values below zero, indicating potential benefit in reducing viral load. Evidence for the potential benefit of the lottery prize vs. fixed compensation was strongest, as most of the interval for the lottery prize was below zero (95% CI: −0.67–0.14). Evidence for the potential benefit of the TMQQ component was weakest, as most of the interval was above zero (95% CI: −0.07–0.76).

### 3.8. Developing research questions for the qualitative analysis

The sequential explanatory mixed methods design is a two-phase process where quantitative data are collected and analyzed first, then qualitative data are collected and analyzed based on the quantitative results (72). In the present study, quantitative and qualitative data were collected concurrently, but the research questions for the qualitative aspect of the study were developed after quantitative analyses were complete, so the qualitative data could be used to add richness, meaning, and context to the quantitative findings. To develop the research questions, the interpretive community comprised of members of the research team met to review and interpret quantitative results and articulate questions that could plausibly be answered by the qualitative data. Then, in the interest of parsimony, we selected two research questions to address in the present study. First, we found in the quantitative analyses the S-CAP2 intervention components were acceptable to participants and feasible, as noted above. However, we did not know participants' views on which aspects of the intervention components were useful, whether for supporting HIV management or other behaviors. This included perspectives on fixed compensation or the prize received for viral suppression, given quantitative results presented above. Second, we wished to examine how the COVID-19 pandemic affected HIV-related health decisions and behaviors during the study. Other research questions identified in this process were determined to be outside the scope of the present study, but could be addressed in future qualitative or mixed methods research.

### 3.9. Overview of qualitative results

As noted above, the present study was carried out in the early stages of the COVID-19 pandemic in a major COVID-19 epicenter. Overall, participants discussed both ongoing multi-level challenges to HIV management, along with the complexity of managing feelings of anxiety and helplessness related to COVID-19, the general uncertainty of daily living during this time, and the ways in which COVID-19 affected their capacity to manage their HIV treatment. Most participants in the present study had been living with HIV for many years. Although participants did not evidence HIV viral suppression at the time of enrollment, the majority were taking HIV medication at some level prior to enrolling, and most of those who were not on HIV medication at the time of enrollment had taken HIV medication within the last 6 months. Thus, they could be considered long-term HIV survivors, and as such had extensive prior experiences taking HIV medication and engaging in HIV care. Quantitative data highlighted that participants were located in the lower socio-economic strata, and less than half the sample was stably housed. Therefore, we addressed the qualitative research questions in the context of the COVID-19 pandemic, the experience of long-term HIV survivorship, and chronic poverty. In the next section we provide an overview of the qualitative study findings, followed by results for the two qualitative research questions and a joint display (Table 5) summarizing the integrated findings.

**Table 5:** Joint display summarizing the integrated results.

**Domain**	**Quantitative results**	**Qualitative results**	**Findings were concurrent, complementary, or discrepant, and comments**
Feasibility	Components and assessments were feasible Having participants obtain their own lab reports from HIV care settings was feasible but challenging (e.g., because participants lacked smartphones and had lower levels of technical abilities). This was complicated by the COVID-19 pandemic. These challenges reduced the number of lab reports provided for analysis.	Compensation for study visits was the main reason participants initially enrolled in the study, but they continued in the study at high rates mainly because of their positive experiences in the study. Compensation overall led participants to feel respected and valued, and this, in turn, promoted retention and engagement.	Qualitative and quantitative results were complementary. Compensation is likely necessary but not sufficient for retention and engagement. The quality of the participant experience drives feasibility. The ClinCard system we used to provide compensation virtually and quickly likely played a role in participants' positive study experiences. We recommend similar studies support participants in obtaining lab reports, and if possible, provide multiple ways for them to do so with minimum burden and hassle, along with cell phones.
Acceptability overall	The study was acceptable to participants overall in a number of respects (e.g., information was helpful, privacy was respected, needs pf racial/ethnic group were understood). Overall acceptability dropped from 82 to 69% at the second FU. Almost all noted they would continue to take HIV medication after the study ended.	Project was acceptable overall. Qualitative results rarely highlight negative or unacceptable experiences in the study.	Lower rates of acceptability at the second FU suggest some participants may not have gotten their needs met in the study. Qualitative results may over-estimate acceptability since those with less positive experiences may decline to be interviewed or to comment.
Evidence of efficacy on viral load and suppression	Viral load levels decreased, and rates of viral suppression increased at FU1 and FU2. This suggests some or all of the components may be “active.”	Some participants discussed ways the project fostered the desire and ability to take HIV medication at higher levels and achieve HIV viral suppression, as well as barriers they experienced. Not all participants wished to achieve viral suppression at this time.	Findings were congruent, with qualitative results perhaps presenting a more favorable view of the effects of the components compared to quantitative findings.
Financial rewards (overall)	NA (see below)	We found both fixed compensation and lottery prizes enhanced positive experiences related to study participation, were not seen as coercive or pressuring, and were seen as a form of encouragement to achieve viral suppression, but not necessarily a primary motivating factor or reason in and of themselves to achieve viral suppression. There were three themes: the financial reward was sufficient to motivate changes in HIV medication adherence behavior, the reward was appreciated but not necessarily a primary motivating factor or reason to achieve viral suppression, or it increased desire to achieve viral suppression but this was not sufficient to overcome serious structural and individual-level barriers to HIV medication adherence. Both levels of this component are acceptable, and feasible if participants provide lab reports and present for FU.	Financial rewards require relatively less emotional and cognitive effort for participants compared to counseling components. Financial rewards require relatively less effort for staff compared to counseling components. Financial rewards for viral suppression hold promise but the type (fixed vs. lottery), amount, and timing warrant further study.
Lottery prize	Feasibility was high since the component is not labor intensive to administrate. At FU1, 62% said the prize prompted them to achieve HIV undetectable viral load, dropping to 43% at FU2. Almost all intended to continue to take medication after the prize was received. Approximately 20% achieved HIV viral suppression at FU1 and 40% at FU2. CIs indicate the lottery prize was the most promising of the component levels with respect to reducing HIV viral load.	The lottery prize appeared more interesting, memorable, and engaging than fixed compensation, consistent with behavioral economic theory. See above re: themes related to the effects of financial rewards on behavior.	Quantitative and qualitative findings are largely congruent: prizes can encourage or support behavior but may not be a primary motivator. It is possible the effects of prizes operate largely outside conscious awareness, consistent with behavioral economic theory. Qualitative results provide insights into the ways the prize was seen and its effects. Taken together, findings suggest the lottery prize may be more promising than fixed compensation for viral suppression, consistent with behavioral economic theory.
Fixed compensation	Equivalent to findings for lottery prize with respect to acceptability and feasibility. Approximately 30% achieved HIV viral suppression at FU1 and 25% at FU2.	See above section on financial rewards.	See above
TMQQ	Feasibility was high: 82% answered at least one QQ and on average responded to 11 of 21 questions. Acceptability was modest (< 70%). Approximately 25% achieved HIV viral suppression at FU1 and 30% at FU2, with no difference between those who received TMQQ or not. In addition to influencing viral suppression, this component was intended to foster engagement in the study. Quantitative results suggest this component had the smallest effect on reducing HIV viral load, but may be useful as a low-touch engagement tool.	TMs were seen as informational, but the information provided was quite basic. Some were frustrated by how easy the QQs were. But, participants reported it “felt good” to get the answers correct. Frequency of TMQQs was acceptable and more frequent TMQQs would be acceptable and feasible. TMs were not necessarily connected to motivation to take HIV medications. TMQQs became reminders to take HIV medication that day for some. The information about HIV was found to be interesting and useful by most. TMQQs were commonly experienced as a form of positive connection with the study. TMQQs were experienced as an aspect of the participant's overall, generally positive, relationship with the study.	Qualitative findings add rich description and context to understanding this component, which was intended in part to foster engagement in the study in the period during which participants might seek to achieve viral suppression. Reminders of HIV status and HIV medication can induce negative feelings. The TMQQs did not appear to do so. Thus, providing short, easy, and private intervention content that does not directly refer to the need to take HIV medication that that yielded compensation may induce positive emotions, or at least no negative emotions. This may be valuable and may support study engagement over time. Findings suggest this component is promising and warrants further study, including regarding more challenging questions and/or individualized messages, and more frequent messages. The TMQQ component requires relatively less emotional and cognitive effort for participants compared to counseling components. Quantitative questions may need refinement to better assess perspectives on this component.
MI counseling sessions	The component was feasible: 95% completed all three sessions, despite sessions being virtual and problems with phones being common. It was also highly acceptable and participants reported it influenced their decisions to take HIV medication (Table 3). Approximately 25% achieved HIV viral suppression at FU1 and 33% at FU2, with no large difference between those who received the component or not.	Highly acceptable and associated with insights and various types of behavior change, such as substance use challenges. It is not clear whether the habit-formation aspect of this component was useful.	Few MI interventions have been carried out virtually (on the phone, not a Voice over Internet Protocol) and during COVID-19. Participants had very high levels of motivation at entry into the study, which may have reduced the need for this component. It is possible serious structural and individual-level barriers to HIV medication adherence including those related to COVID-19 impeded behavior change even when sessions were provided.
Motivation (mediator of component effects)	Motivation for HIV viral suppression is high at study enrollment (~90/100).	Motivation was assessed indirectly in the qualitative results (e.g., goals for viral suppression). Some components increased motivation for viral suppression, as noted above, but in many cases, motivation was not sufficient to overcome serious structural and individual-level barriers to HIV medication adherence.	We cannot explain with precision why motivation for HIV viral suppression is very high at enrollment (although it may be related to COVID-19), but participants were not virally suppressed (despite taking HIV medication in some cases) and many did not achieve viral suppression during the study. There is a large literature on multi-level barriers to viral suppression, but we do not yet understand participants' perspectives on this phenomenon.
Diverting (selling) HIV medication	Not assessed	Diverting HIV medications is common and a power structural impediment to achieving HIV viral suppression for some.	Pharmacies eliciting illegal medication diversion were a serious barrier to HIV management for some.
Effects of COVID-19	Not assessed	COVID-19 overall created impediments to HIV management but also increased motivation to manage HIV in some cases.	Understanding COVID-19 as a contextual factor was vital to interpreting study findings.
How to improve procedures and components	Some measures may need refinement for more precise estimates of effects.	Various improvements to components and study procedures (regarding laboratory reports) were identified.	Having both quantitative and qualitative data and integrating results was useful. Quantitative data captured experiences of participants as a whole and qualitative data provided richness, detail, and context but seemed skewed toward more positive experiences with the study. Quantitative data required us to not over-estimate the positive aspects of participants' experiences.

Participants discussed a number of significant barriers and interruptions to healthcare access in response to COVID-19, which in turn interfered with their sustained HIV medication use. As we describe throughout this section, some of these barriers pre-dated the COVID-19 pandemic, some were exacerbated by COVID-19, and others were specific to COVID-19. Further, the COVID-19 pandemic certainly served as the backdrop for how the intervention components were experienced with respect to promoting HIV care engagement. In exploring the disconnect between reported high levels of motivation for HIV medication adherence and viral suppression found in the quantitative results, common barriers to HIV management reported by participants included structural impediments such as financial insecurity, housing instability, safety concerns within housing circumstances, and other factors that limited material resources. Importantly, participants underscored the cumulative, synergistic effects of these and other related structural barriers on their mental health and/or substance use patterns, which in turn, frequently diminished HIV treatment as a priority in participants' lives. Some participants expressed complicated perspectives about their individual HIV care, often affected by their state of wellbeing, but also the pressure they felt to attain viral suppression, particularly during periods of elevated stress, which, in turn, exacerbated feelings of stress.

Chronic poverty contributed to some participants selling (or diverting) their HIV medication doses to pharmacies operating outside the law, an opportunity to receive financial resources that participants typically found very challenging to decline. Study findings made evident aspects of the intervention components that were experienced as positive, as described below. Findings demonstrated key aspects of the intervention components that were useful in supporting HIV management, as well as ways the intervention components could be improved. One goal of the study was to allow participants to engage in the components even if they could not or did not achieve HIV suppression, and we attended to positive effects of components on behaviors other than HIV management. Names used below are pseudonyms and identifying details have been removed or obscured to protect participants' confidentiality. Participants were queried about their preferred pronouns (e.g., he, she, they, other) at enrollment, and the appropriate pronoun is used below in the description of each participant.

### 3.10. RQ1: what aspects of the S-CAP2 intervention components were useful, including in supporting HIV management, and how could they be improved?

#### 3.10.1. Overview of results for RQ1

Despite the structural and other challenges to HIV management that were common among participants, including related to COVID-19 as described in more detail below, the majority viewed engagement in the S-CAP2 intervention components as markedly beneficial to their individual wellbeing, whether they achieved HIV viral suppression or not. In particular, supportive and nonjudgmental interactions between study team members and participants often prompted participants to reflect on and, in some cases, reconsider or even modify their personal attitudes toward and behaviors regarding HIV medication adherence. In the sections that follow we describe participants' perspectives on each of the three intervention components, with an emphasis on the ways each component influenced HIV management, and other factors that may have contributed to the satisfactory-to-high levels of acceptability and feasibility as described in the quantitative results.

### 3.11. Component A: financial reward for viral suppression, and compensation in general

As noted above, participants received compensation for study activities such as assessments and providing laboratory reports, and all participants could receive a financial reward if they achieved HIV viral suppression at the first FU assessment: fixed compensation ($300) or a lottery prize (maximum prize $500). Those who did not achieve viral suppression received $50. Overall, compensation for study activities was viewed as highly acceptable among participants, was the primary reason they joined the study, and for many, led them to feel respected and valued in the study (“They [S-CAP2] don't give you pressure, they give you encouragement. [Compensation] did play a role as part of the encouragement I felt. Because I feel like they understood the fact that you don't live life for free and your time is valuable”). Compensation in general was described as promoting study engagement and participants commonly reported that it, along with a positive experience with staff and study activities, encouraged them to remain active for the duration of the study. We found both fixed compensation and lottery prizes enhanced these positive experiences related to study participation and were not seen as coercive or pressuring.

Participants' views on the attitudinal and behavioral effects of financial rewards could be organized into three main themes: in some cases, the financial reward was seen as sufficient to motivate changes in HIV medication adherence behavior (that is, serving as “nudge” and a source of hope and encouragement), and in other cases, it was appreciated but not necessarily a primary motivating factor or reason to achieve viral suppression. Third, some participants reported the financial reward increased their motivation to achieve viral suppression, but this motivation was not sufficient to overcome serious structural and individual-level barriers to HIV medication adherence. Qualitative results did not yield evidence of any major differences in participants views on financial rewards based on whether they received fixed compensation or a lottery prize, although the lottery prize came across as more exciting and memorable than fixed compensation. Regarding the first theme where the financial reward served as a nudge, Bryant, a 40-year-old cisgender, heterosexual Black man who was diagnosed with HIV < 10 years ago, was assigned to an experimental condition that included the lottery prize and noted:

It encourages me to reach the goal of becoming undetected and also just a reminder. Like listen, you know, I should stay on taking my meds every time I should be taking them.

Mark was a 60-year-old cisgender, heterosexual Black man who as diagnosed with HIV 30 years ago, who was also assigned to an experimental condition that included the lottery prize. He did achieve viral suppression, and won the largest possible prize. While the funds were appreciated, and the experience of spinning the prize wheel was exciting, he noted that the prize was not the actual reason for his change in behavior.

She spins the wheel, you can hear it. […] So, listen to it. So, she's spinning it and I'm hearing it. I kept saying, “Oh man, I want to win this money. I want to win this money.” She says, “Mr. [name redacted], you won.” I was like, “Thank you.” But I'm not doing it for the money. […] I try not to look at it like that. I try to look at this as a beneficial thing. It's a helpful thing for me. Like you all kept me on track with this, and I'm just continue on being on track, you know, when the study is over. You know, I'm continue on, you know, staying on track with it. I'm not doing this for that. You know, I'm doing it to benefit me health-wise, not financial-wise. Health-wise, you know, because money doesn't mean anything for me. You know what I mean? I grew up poor. We didn't have anything. So when, you know, when I get a little few dollars every now and then, I put it toward my apartment or I treat myself or something or I help somebody out that's less fortunate.

Mark continued:

It wasn't about the money, it was about the hope that you all gave me, the encouragement, man, to stay on time and take the medications on time. You know what I mean? Because, you know, for a long time, I used to get real depressed, being detected [having detectable viral load].

Jason, a 35-year-old cisgender, gay Black man, described a 20-year history living with HIV during which he had achieved viral suppression only once, in part because of concerns about HIV medication side effects and because he sold his medication to meet financial needs. Yet, during his time in the study, he seriously considered taking HIV medication, and began to explore his fears about the adverse health consequences of not doing so. The chance to win a lottery prize increased his motivation for viral suppression, but ultimately, financial constraints prevented him from doing so. Yet, he also believed that if he achieved undetectable HIV viral load, he would continue taking HIV medication.

I have a serious issue with the medication as a whole, regarding long term. Short term is great. Long terms is not so great to continue to that medicine in your body once you have leveled off and get the virus out of the body due to the fact of the kidney and the liver damage. […] I can tell you right now, I actually have a desire to take my meds. I'm currently trying to get over this mental health hump and financial crunch. […] The fact of I need to take my meds or I'm going to die. Or it's either choosing to take the meds and stay financially twisted, because that $350– that's my biggest check, is the medicine I sell. […] That final end to the bonus of possibly getting $500 for the undetectable almost made me take my bottle. I'll pop the pill, because I knew, I know if I pop the pill in about 40 days my body bounces like right back [to undetectable viral load]. […] I almost did it. But then—I did it but then I didn't. […] It's all finances for me.

Thus, Jason highlights the complexities of HIV management in the context of chronic poverty.

In summary, the COVID-19 pandemic intensified existing financial strains for many participants. Study compensation was a release valve for financial pressures for most and even offered positive experiences during periods of sustained stress. The financial reward component was acceptable to participants, no adverse effects were detected, and the component influenced attitudes and behaviors in a number of ways.

### 3.12. Component B: TMQQs

In the present study, TM reminders were paired with QQ each week to assess knowledge gained and to generate points for which participants earned compensation. Participants reported TMQQs were appreciated, it was generally enjoyable to answer the QQ and receive correct answers, and the TMQQ component served a number of functions. First, those currently taking HIV medication experienced the TMQQs as a reminder to take HIV medication. Although HIV knowledge was relatively high for the majority of respondents, particularly those who had lived with HIV for a decade or more, TMQQs served a function of keeping HIV medication adherence in the foreground of their daily activities and decisions, even though the TMs did not focus on medication or adherence. (“Yeah, it's just a constant reminder, the constant influx of information flying in someone's phone…I think it's a great reminder.”). Second, the information provided about HIV was found to be interesting and useful for the most part. Third, the TMQQs were commonly experienced as a form of positive connection with the study. Overall, TMQQs were seen as fostering study engagement.

Participants noted TMQQs could be improved in two main ways: they could be more challenging (as they were too easy in most cases, since participants were generally expert on HIV) and should be sent more frequently than once a week. Nonetheless, one of the aims of the TMQQ component were to support continued engagement, and as such they were not intended to be difficult to answer. In most cases, TMQQs were not associated in participants' minds with *changes* in motivation to take HIV medication with high levels of adherence, but in some cases the reminders did support existing adherence patterns (“At first, I was undetectable, but then I started reading those text messages and one of them came in one week and that one motivated me to just continue to take the medication”). As Jason, introduced above, noted:

Would [TMQQs] get people to take their medicine? No. No, I think—I'm not sure it would get people to take their meds. I just think it would be a little bit more challenging. For someone like me, when I saw those questions, I wasn't learning anything. You've got to think about the people that have been sick for a little bit and know the knowledge. So I didn't learn anything new on the questions. It was just a reminder to take my meds. So asking the questions became reminders. I guess that's the first thing I—and that really, I could say that that's cool. The questions did become reminders. […] The fact that the questions were coming to me and things, it keep the HIV medication thing in my head all the time.

Participants reported that receiving HIV-related information, such as in medical settings, was not typically associated with positive emotions, and was commonly avoided. However, the TMQQs were a positive experience for most. This was largely related to the fact that information was brief and limited, received by text message, compensation was provided for correct responses on the TMQQs, and TMQQs were associated with the larger generally positive experience of engaging in the S-CAP2 study.

### 3.13. Component C: MI counseling sessions

As a reminder, all intervention components were informed by the conceptual model that incorporates critical race theory and that includes autonomy support and harm reduction and aligns with MI, but the counseling session component also used specific MI techniques such as identifying discrepancy, a “readiness ruler,” and training on habit formation. Participants described themselves as knowledgeable about HIV and reported they did not require training on how to manage HIV. In general, MI counseling sessions were experienced as acceptable and useful in that they provided a supportive and non-judgmental experience in which to reflect on behavior patterns. Importantly, this commonly prompted participants to reflect on and/or reconsider their attitudes toward HIV medication adherence and adherence patterns. The supportive, non-judgmental approach engendered participants' feelings of connectedness with the S-CAP2 study while also centering participants' individual health needs, which may or may not have included HIV medication adherence and HIV viral suppression. Further, the sessions allowed for open and honest sharing of their experiences, which further promoted participants' health decision making. This was notable, because participants commonly reported concerns or fears about discussing potentially stigmatized issues such as declining to take HIV medication, along with behaviors such as drug use, selling medications, and other “hustles” in the context of a research study (“I've been selling my medication for so long. I know that this is all confidential, right? Right?” and “I'm being pretty open with you and because, what are you going to do? I'm [not] going to see helicopters over my roof tomorrow, I mean”). Ultimately, participants were clear they made their own decisions about HIV management, consistent with the MI approach. Further, as noted above, taking HIV medication was not a precondition for study participation.

One participant who had been living with HIV for over 20 years shared how MI counseling sessions helped maintain a focus on his health. In sessions, the participant shared that, within the context of the staff member-participant relationship, he engaged in self-reflection, and was open and honest with the interventionist, which, in turn, appeared to have played a role in his continuing to avoid heavy drug use and begin to take HIV medication with high levels of adherence (“I'm trying to be undetectable as before”), in conjunction with improvement in his housing placement. Daniel, a 56-year-old cisgender, heterosexual Black man diagnosed with HIV 20 years ago, described:

Well, [MI Counseling Sessions] helped me to stay focused on my physical health. You know what I'm saying? My mental health, as far as me, you know, living, living with a medical condition, the one I have. […] I'm not going to be in denial; you know, 20 some years I've been medically disabled. So I look at it like this. If I live this long, I can live longer. Just keep focused, pray, ask God to help me. You know, take my medication, do the right thing, and stay off the drugs. You know what I'm saying? I drink here and there, I smoke a little weed. I don't do all that other shit. You know? I've been there, done that. You know, them days are over with. […] [With staff member], it was like I talked to her like a sister. Anything she wanted to know, I tell her. Whatever's going on in my life, I kick it to her. You know, the only way to get answers, get help, you've got to be honest with yourself. […] Right. I can trust her.

Importantly, staff and participants were generally able to construct productive working relationships, despite being unable to meet in-person due to COVID-19, as described by Daniel.

A typical constellation of serious barriers to HIV medication adherence for participants comprised persistent mental health issues, substance use challenges, and sub-optimal housing, which, not surprisingly, reduced HIV care and HIV medication as priorities in their lives and interfered with participants' capacity to effectively manage their HIV treatment. MI counseling sessions, which were flexible and individualized and could attend to such barriers along with HIV care engagement and HIV medication served the purpose of supporting participants in centering or re-centering wellbeing along with HIV-related health, with the potential to interrupt periods of distress, heavy substance use, and/or sub-optimal HIV management or solidify a commitment to behaviors and relationships that could support wellbeing. Mary, a 54-year-old Black heterosexual, cisgender woman diagnosed with HIV 25 years ago, described the importance of an improved housing situation in her HIV management, and the role MI counseling sessions played in her articulating and carrying out her own health-related decisions.

[I stopped taking HIV medication] because of depression, my mother passing away, me moving from shelter to shelter. And then when I do get the medication at times I just say [forget it] you know? And now I'm staying […] in one place now and I know I'm not going nowhere no time so, yeah. […] I can get back on my program as far as [taking medication] every time—because I used to wake up in the morning—before I put my feet on the ground, I'd take all my medications you know? [S-CAP2 helped with that stability]. And I said now that I've got my place, I could start back taking my medication like I was taking it. […] You know that's what I choose to want to do? Yes. [S-CAP2] encouraged me and it was me that was encouraged to want to do it, you know?

### 3.14. RQ2: how did the COVID-19 pandemic affect HIV-related health decisions and behaviors during the study?

Participant narratives reflected emotional challenges (fear, loneliness, depression, stress) and clear obstacles to accessing healthcare and social services during the COVID-19 pandemic (“I used to [go to social service center], but now with this Corona nothing is going on. It's horrible. And that made me in a deep depression too because there's nothing to do”). As in many other locations, participants were under stay-at-home orders and physical distancing protocols, which contributed to a disruption in care and services, including medication delivery and drug program groups. Some participants stopped taking HIV medication during the COVID-19 pandemic, and experienced reduced access to substance use treatment and social services. Others increased their HIV medication adherence rate to better protect themselves from the effects of COVID-19. Tracey, a 60-year-old cisgender, heterosexual woman diagnosed with HIV 15 years ago, noted that HIV medication use was abruptly interrupted by the COVID-19 pandemic.

I mean I would take them [HIV medication] if I could. […] Coronavirus came out, which hurt me from having my medical [home health aide] to come out to my house and work with me. They used to come out, [check my] pill box, stuff like that. That would help me a lot. And if they weren't here, yeah it caused a lot of problems. And after that hopefully we'll get back in our regiment and be able to come out to my house and things like that. So, we're working closely, hopefully we'll get this thing settled, and I'll be on my meds regularly.

These disruption to relationships and services, and the tumultuous sociopolitical context in which COVID-19 occurred, commonly had adverse effects on mental health. Participants reported feeling isolated during COVID-19, and recounted reports of social unrest and violence in the news related to the consolidation of the racial justice and Black Lives Matter movements. Thus, the COVID-19 pandemic often led to or exacerbated depression and sadness. Others found in-person therapy and medical visits difficult to manage because of anxieties about COVID-19, especially being around strangers. In fact, concerns about contracting COVID-19 when living with a compromised immune system combined with the fall-out of managing health needs with COVID-19 were palpable among participants. One participant talked about his struggle in deciding whether or not to continue taking HIV medication while sick with COVID-19. He described the shared decision-making process that unfolded during a phone contact with a nurse who followed up with him as part of outreach. The participant, Mark, introduced above, revealed the uncertainty and stress that health decision-making engenders, particularly when living with chronic illness such as HIV.

Well, I had it [COVID-19] back in February, right and when I had it…I had to quarantine in a hotel in New York. My first 3 days being in a hotel and having this COVID, I was like, “I'm not taking this HIV medication. I'm not taking shit.” So, after that day passed, I'm sitting in that hotel room and I'm like literally crying and my mind went back to 1998 and I said to myself, “If I don't take this medication, right, and me having this COVID, my T-cells going to drop.” Because now the COVID is attacking my immune system. I have nothing to fight the virus and I have COVID. A nurse calls me on the phone, and she said, “How you doing, Mr. [name redacted], with the COVID?” I said, “I'm not doing well.” And we talked about HIV medication. She said, “Let me explain something to you, OK? Take the HIV medication because it will help you. Because if you stop taking the HIV medication, the COVID is going to attack your immune system.

The risk of contracting COVID-19 commonly motivated participants to resume HIV medication use, if possible, for fear of the effects of COVID-19 on health while living with HIV. The S-CAP2 study was a source of information for participants to better understand the potential effects of not taking HIV medication. Mark noted how support from a health care professional became the catalyst for improved medication adherence, which helped him reach HIV viral suppression.

In general, participants' narratives demonstrated the challenges of HIV-related health decisions particularly in a disrupted healthcare environment. Although the desire and motivation for HIV viral suppression were generally high, participants discussed how barriers to and interruptions in HIV care may derail their HIV management efforts, but also how they often reprioritized HIV medication and their HIV care with ongoing support and encouragement, including through the S-CAP2 project activities.

## 4. Discussion

The goal of ending the HIV epidemic in the United States cannot be reached without addressing the complex impediments that African American/Black and Latino PLWH experience to consistent engagement along the HIV care continuum. Clearly, efficient and effective behavioral interventions are an essential aspect of supporting these populations of PLWH in making use of and benefitting from HIV care and medications (73). The present study seeks to advance the portfolio of low-touch and virtual behavioral intervention approaches for the large and growing population of African American/Black and Latino PLWH, the vast majority of whom are located in the lowest socioeconomic strata. As we describe above, this population of PLWH experiences barriers to the HIV care continuum at structural, social/cultural, and individual levels of influence, and these barriers also can impede their participation in research (74, 75). Thus, they are under-studied compared to PLWH well-engaged in HIV care settings (74, 75). Further, since 2020, the COVID-19 pandemic has complicated HIV management in many locations (16). The present study was carried out in the relatively early stages of the COVID-19 pandemic when PLWH experienced disruptions to access to HIV care and services, social relationships, and their livelihoods. Further, the COVID-19 pandemic precluded in-person activities with research participants at our institution during this time and study activities were carried out over the phone or virtually, since participants rarely had computer or smartphone access (19).

We apply the MOST framework and use an efficient factorial design to explore three behavioral intervention components directed at African American/Black and Latino PLWH with non-suppressed HIV viral load. All three components are delivered virtually, and two of them (TMQQ and financial rewards for viral suppression) are low-touch and require minimal staff time to administrate. The factorial design used in the present study allows for a more precise understanding of the components' acceptability, feasibility, and preliminary evidence of effects compared to the classical approach of testing multi-component “packaged” interventions in randomized controlled trials. This factorial design, combined with the mixed methods approach, produced findings that advance research on low-touch and virtual interventions for American/Black and Latino PLWH with serious barriers to the HIV care continuum. In particular, the sequential explanatory mixed-method approach provided vital richness, depth, and context to the study results, and also enhanced the study's validity (72, 76). The present study highlights the acceptability, feasibility, and in some cases, preliminary evidence of effects of novel behavioral intervention components to improve HIV self-management for this population at-risk for poor HIV outcomes, and suggests directions for the next stage of this program of research.

The challenges inherent in managing HIV in the context of structural violence, including chronic poverty, cannot be overstated, as the present study highlights. Structural violence is defined as the social forces that harm certain groups of people, producing and perpetuating inequality in health and wellbeing (77). Consistent with past literature, financial insecurity, lack of financial and material resources, housing instability, and safety concerns within housing circumstances are common barriers to HIV management among African American/Black and Latino PLWH (78, 79). We found these types of barriers appear to function in cumulative and synergic ways to thereby exacerbate mental health and/or harmful substance use, which then generally diminishes HIV self-management abilities. In addition, participants commonly describe being approached by pharmacies that sought to purchase their HIV medication from them, called medication diversion (80). These actions by pharmacies are illegal, and certainly a serious impediment to PLWH with financial constraints, as immediate material needs will generally take priority over longer-term health outcomes (81). The present study was carried out in this challenging context.

As noted above, and contrary to expectations, participants' motivation to reach HIV viral suppression was high at the time they were enrolled in the study and increased during the study. In our previous study, motivation for high HIV medication adherence was somewhat lower at enrollment (Mean = 73.1 on a 0–100 scale, SD = 26.6) (11). It is possible that motivation for HIV viral suppression was elevated in the present study due to the COVID-19 pandemic, which triggered health concerns for many PLWH. Yet, none evidenced viral suppression at the time of enrollment. This may be due to the severity of structural barriers to HIV management and their downstream effects on mental health and substance use management, both long-standing barriers and those related to COVID-19. The factors that impede PLWH reaching HIV viral suppression even when motivation to do so is very high warrants further study, along with how such factors operate and how they can be ameliorated. While the present study sought to identify and circumvent some structural barriers to HIV care, clearly, mitigating structural violence would have powerful positive downstream effects on HIV management (82, 83). Such efforts could include universal basic income, improving housing quality, and providing easy access to high-quality HIV care (84–87).

While reaching HIV viral suppression may be difficult in the context of structural violence, and complicated by the COVID-19 pandemic, behavioral interventions are clearly an essential aspect of ending the HIV epidemic (84). And, a substantial proportion of participants during the present study (up to ~40%) moved from unsuppressed to suppressed viral load. As noted above, the present study focuses on a population of PLWH who reside in high-risk contexts and are not well-embedded in medical settings. In fact, many behavioral intervention studies in the field of HIV focus on assisting participants who are initiating HIV medication with achieving HIV viral suppression (88), those at risk for stopping medication (89), or are focused on strategies to allow those who evidence viral suppression to sustain it (90, 91). Fewer studies focus on the population of PLWH examined here: mainly long-term HIV survivors who do not evidence HIV viral suppression and who experience serious barriers to engagement along the HIV care continuum (92). Rates of viral suppression reached among participants in the present study are comparable to past studies with similar subpopulations of PLWH (93, 94), and, notably, the intervention components in the present study were virtual and/or low-touch, suggesting the promise of cost-effective interventions for this population that faces serious challenges.

The field of behavioral economics, including the study of the types, magnitude, duration, and timing of conditional economic incentive approaches to supporting HIV management and “nudging” participants toward their personal health goals, are relatively new areas of study. In general, financial incentives work best when applied close in time to the desired behavior (95, 96). Designing effective financial rewards for HIV viral suppression is complicated by a number of factors including that self-reported adherence may not be sufficiently accurate to allocate rewards, laboratory testing for viral suppression is not usually done on a frequent basis, and PLWH must take HIV medication at high levels for months before reaching suppression (97). Nonetheless, past research has highlighted the utility of providing a financial reward for viral suppression (98, 99). Our own past S-CAP study suggested that lottery prizes were acceptable and feasible, but that some participants might have preferred a fixed level of compensation (11). However, these two types of financial rewards had not yet been directly compared.

The present pilot study provides evidence for the acceptability and feasibility of both types of financial rewards, and we did not find any social harms or negative effects related to this component. About half the participants said the financial reward played a role in decisions about HIV medication or led them to try achieving undetectable viral load, regardless of whether a participant was assigned to the fixed compensation or lottery prize. Qualitative results were consistent with behavioral economic theory in that PLWH generally did not experience the financial reward as directly causing their decisions and behavior. But, the financial reward could align with participants' intrinsic motivation and goals to support HIV management behavior, consistent with behavioral economic theory (100, 101). Further, financial rewards and compensation in general were experienced by participants as a form of respect for their time and contribution to the study, or even a form of emotional support in some cases (“It wasn't about the money, it was about the hope that you all gave me, the encouragement … to take the medications on time.”) Qualitative data suggest that the use of a prize wheel for the lottery prize was engaging, exciting, and memorable in comparison to the fixed compensation (i.e., made the receiving the reward more ‘salient'), consistent with behavioral economic theory (100, 102, 103). The financial reward component may have promoted engagement in the study, and did not require extensive staff time to administer. Further, the financial reward did not “crowd out” intrinsic motivation for health, again consistent with behavioral economic theory (100, 102, 103). Overall, study findings indicate that financial rewards for HIV viral suppression hold promise for this population of PLWH, particularly lottery prizes, and yielded many important research questions regarding the optimal type, size, duration, and timing of financial rewards for HIV viral suppression, along with how to sustain behavior change after rewards conclude (98).

The TMQQ component was automated and intended in motivate viral suppression, to support engagement in the study during the period where participants might increase HIV medication use to reach viral suppression, and to foster study retention. The feasibility of this component was reasonable. Acceptability was moderate in quantitative results and appeared high in qualitative results, highlighting the utility of the mixed methods approach for enhancing validity of findings. One major factor affecting this component was participants' severe financial hardship, which interfered with consistent access to a working cell phone that could receive the TMQQs. Thus, many participants wished to complete the TMQQ component but were unable to, reducing participation rates. In some cases, the TMs were experienced positively, perhaps as a form of connection to the study, and served as a reminder to take HIV medications. We found it is challenging for research teams, even in collaboration with a Community Advisory Board, to create TMQQs with the right level of difficulty for participants. Although as an engagement tool, the QQs were not intended to be difficult, some participants reported the TMQQs were too simplistic given their extensive experiences living with HIV, suggesting that advanced HIV information is needed and welcomed. It is possible that allowing participants to choose between a set of basic vs. advanced messages would have utility in future research, along with providing cell phones, as we note below. Alternately, messages could vary in level of difficulty, and participants can receive more difficult messages if they respond correctly to easier ones.

As noted above, the intervention components are grounded in a conceptual model that incorporates critical race theory and that includes autonomy support and harm reduction. Participants' views on the MI counseling session component were consistent with the elements of this model. For example, they reported the component provided an opportunity to reflect on and/or reconsider their attitudes toward HIV management and the non-judgmental, pressure-free approach that stimulated participants' feelings of connectedness with the study. The MI sessions component was highly acceptable and feasible, even virtually and carried out over the phone. Most participants assigned to the MI counseling sessions component said in the acceptability ratings that the sessions played a role in their HIV medication decisions and led them to increase HIV medication adherence. Most participants assigned to this component also said the MI counseling sessions led them to try achieving undetectable HIV viral load. However, quantitative results (Table 4) did not reflect these findings.

MI is designed in large part to strengthen personal motivation for and commitment to a specific goal by eliciting and exploring the person's own reasons for change within an atmosphere of acceptance and compassion (33). In the present study, as noted above, motivation for viral suppression was very high at the time participants enrolled in the study and increased, and was somewhat higher at enrolment than in past studies, perhaps in part related to the COVID-19 pandemic. Yet, despite this high level of motivation, the majority of participants did not reach viral suppression during the study. This suggests the main aspects of MI—to build durable, intrinsic motivation for behavior change—were not salient for most participants in this particular context. This finding contrasts with the large literature on the effects of MI interventions in a range of contexts and for a variety of health outcomes, as described above.

These findings have implications for the design of this MI component: it should be flexible and individualized to spend little time on strengthening personal motivation for change in cases where such motivation is strong, and more time on uncovering, understanding, and resolving barriers to related behavioral concerns such as mental health treatment and substance use patterns. Indeed, these were important barriers to viral suppression in the present study and MI approaches are effective with such behaviors (104). The MI sessions component included a focus on habit formation, but results did not indicate whether participants found this useful. However, counseling intervention components are costly to administer compared to low-touch components (105), suggesting the need for future cost-effectiveness analyses. These findings provide support for a fully-powered efficient factorial design that can both ascertain the potential of intervention components in both implementation and cost-effectiveness.

As interventionists, in addition to exploring acceptability and feasibility in early-stage research, we are interested in whether intervention components “work.” The main purpose of conducting a pilot study such as this pilot optimization trial is to examine the acceptability and feasibility of an approach that is intended to be used in a larger scale study (63, 106). But, the efficacy or effectiveness of interventions cannot be evaluated in a pilot study because of insufficient statistical power to detect effects (63). On the other hand, it is challenging to move a program of research from a pilot study to larger scale studies without some “signal” that the interventions tested show promise. So, despite constraints related to low statistical power, we can look for preliminary evidence of effects of components, as we have done here, a type of “non-futility.” (107). In other words, we have explored whether any of the components show evidence of effects, with the understanding that the lack of effects does not necessarily mean the component is futile or completely inactive. We examined confidence intervals for each component's main effect on log_10_ viral load at FU2 and found that none of the components would be considered futile, but that the TMQQ component had the weakest effect on HIV viral load and may not be an optimal component for improving viral suppression in this population. Nonetheless, the TMQQ component appears to have utility as an engagement tool. The study findings taken together, and considering the larger literature, suggest that all three components warrant further study and that, consistent with behavioral economics theory, financial rewards in the form of lottery prizes may hold greater promise than fixed compensation of similar value.

## 5. Limitations

Strengths of this pilot optimization trial include the factorial design, mixed methods approach, and evaluation of the primary outcome by laboratory report, an objective measure. It also has limitations. The sample did not include monolingual Spanish-speaking participants, which limits the generalizability of study findings to the population of Latino PLWH as a whole. The sampling method and study procedures (such as the requirement for participants to provide their own laboratory reports and to have their own phones to receive TMQQs) may have introduced bias by reducing participation rates among those PLWH with the greatest barriers to HIV care and the least material resources. Further, inconsistent access to cell phones appeared to reduce participation in the TMQQ component. In future research, we can help reduce these potential biases by providing HIV viral load testing and cell phones to participants as part of the study. We will also explore factors that predict higher vs. lower levels of participation in the TMQQ component. Last, since African American/Black and Latino PLWH tend to stop and start HIV medication, as shown in past research (12), a proportion of the sample could be expected to reach HIV viral suppression during the study for factors unrelated to the intervention components. A fully-powered trial is needed to estimate the effects of each intervention component with precision. Results must be interpreted within the context of the COVID-19 pandemic and may not generalize to other contexts.

## 6. Conclusion

A range of intervention approaches are needed for African American/Black and Latino PWLH who are poorly or inconsistently engaged along the HIV care continuum in order to achieve the public health goal of ending the HIV epidemic. In particular, lower-touch interventions that reduce the burden on health care systems and that can be carried out virtually are needed (108, 109). The relatively low-touch intervention components grounded in behavioral economics and MI tested in the present study are acceptable and feasible and warrant further refinement and study in future research. Further, these approaches can be carried out outside the traditional health care settings to which this population of African American/Black and Latino PLWH experiences serious barriers. The MOST framework and the factorial design have an important role to play in the development of future efficient multi-component interventions.

## Data availability statement

The raw data supporting the conclusions of this article will be made available by the authors, without undue reservation.

## Ethics statement

The studies involving human participants were reviewed and approved by Institutional Review Board, New York University. Written informed consent for participation was not required for this study in accordance with the national legislation and the institutional requirements.

## Author contributions

PF, MG, CC, and SL conceptualized the purpose and design of the present study, contributed to the data analysis, and wrote much of the manuscript. CC was the principal statistician. SS directed the study and served as a member of the interpretive community that analyzed, interpreted, and integrated data. CR-D was a study co-Investigator and advisor on study procedures. RF and SC were the main qualitative methodologists and analysts, and served as leaders of the interpretive community. SC and KI carried out study procedures and intervention components and critiqued the manuscript. SC was a member of the interpretive community. All authors commented on previous versions of the manuscript and read and approved the final manuscript.
