# Supplementary material for: A virtual pilot optimization trial for African American/Black and Latino persons with non-suppressed HIV viral load grounded in motivational interviewing and behavioral economics
Source: Front Public Health. 2023 May 10;11:1167104. doi: 10.3389/fpubh.2023.1167104 (PMC10205984; doi:10.3389/fpubh.2023.1167104)
Supplement: Supplementary file 1 [file Table_1.DOCX]

| **Supplementary Table 1: Text messages and quiz questions used in Component B** | | | |
| --- | --- | --- | --- |
|  | **Informational or motivational statement (TM)** | **T/F quiz question (QQ)** | **Answer** |
| 1 | Hello from SCAP2, [NAME]. Did you know regular exercise (walking, running, biking, swimming, dancing, yoga, etc.) can help to maintain health and wellbeing? More info here: [URL REDACTED] | SCAP2 True or False question:  ** Regular exercise (walking, dancing, yoga, etc.) can help to maintain health and wellbeing. ** Press 1 for true, Press 2 for false. Respond to the text message to earn points and cash. | TRUE |
| 2 | Hello from SCAP2. We wanted to remind you that it’s never too early or too late to work towards being the healthiest you. Have a great week, [NAME]! | SCAP2 True or False question:  ** It is never too late to work toward becoming your healthiest you. **  Press 1 for true, Press 2 for false. Respond to the text to earn points and cash. | True |
| 3 | Hi from SCAP2, [NAME]. Did you ever take a break from HIV medication? It’s okay! People can still benefit from medications if they decide to start again. | SCAP2 True or False question:  **People who have taken a break from HIV medication can still benefit from medication if they decide to start again.** Press 1 for true, Press 2 for false. Respond to the text to earn points and cash. | TRUE |
| 4 | Hello, [NAME]. Today’s SCAP2 health text message is about smoking. Smoking is very common among people living with HIV. But, unfortunately, smoking is harmful to people living with HIV. Here are some links to programs if you smoke and want to quit. [URL REDACTED] | SCAP2 True or False question:  ** Smoking is not harmful to people living with HIV. ** Press 1 for true, Press 2 for false. Respond to the text to earn points and cash. | FALSE |
| 5 | SCAP2 is thinking about you today, [NAME]! Here’s your health information: Today’s HIV medications are so effective, that if taken with high levels of adherence, people can live a long and healthy life. | SCAP2 True or False question:  ** Today’s HIV medications are so effective, that if taken with high levels of adherence, most people can live a long and healthy life. ** Press 1 for true, Press 2 for false. Respond to the text to earn points and cash. | TRUE |
| 6 | Hi [NAME]! SCAP2 is sending good thoughts your way. Be patient with yourself as you evolve. Small, healthy choices make a big difference in the long run. | SCAP2 True or False question:  **Small healthy choices add up, and can make a big difference in your health in the long run. **  Press 1 for true, Press 2 for false. Respond to the text to earn points and cash. | TRUE |
| 7 | Hello, [NAME]. It’s your weekly SCAP2 text message! Did you know that if your HIV viral load goes down when you start taking HIV medication, it means that the medication is working well? Click the link for more info. [URL REDACTED] | SCAP2 True or False question:  ** If your HIV viral load goes down when you start taking HIV medication, it means that the medication is working well. **  Press 1 for true, Press 2 for false. Respond to the text to earn points and cash. | TRUE |
| 8 | [NAME], hello from SCAP2! Just a quick note to remind you that you can protect the ones you love. If they are not living with HIV, PrEP can help them stay that way. [URL REDACTED] | SCAP2 True or False question:  ** People who are not living with HIV can take pre-exposure prophylaxis (PrEP) so they do not contract HIV. ** Press 1 for true, Press 2 for false. Respond to the text to earn points and cash. | TRUE |
| 9 | [NAME], SCAP2 knows that stress is real. Feel stressed out? Try activities like meditation, acupuncture, and yoga. | SCAP2 True or False question:  ** There is not much you can do about stress, and activities like meditation, acupuncture, and yoga do not help. ** Press 1 for true, Press 2 for false. Respond to the text to earn points and cash. | FALSE |
| 10 | Hello from SCAP2. Always remember, whatever it takes, you are worth it! Eat food that nourishes your body. Get up and move every day. Treat your mind and body with love and respect. Not next month. Not tomorrow. Today. | SCAP2 True or False question:  ** Moving every day and eating food to nourish your body are a few important ways to treat yourself with the love and respect you deserve. ** Press 1 for true, Press 2 for false. Respond to the text to earn points and cash. | TRUE |
| 11 | Hello, [NAME]. SCAP2 is all about drinking a lot of water these days. Did you know staying hydrated can improve your energy and even your mood? | SCAP2 True or False question:  ** Drinking water is not part of staying healthy. ** Press 1 for true, Press 2 for false. Respond to the text to earn points and cash. | FALSE |
| 12 | It’s SCAP2 text message time! SCAP2 has a philosophy: Take care of your health for you, always ignore the haters, doubters, and unhealthy examples that were once feeding you. You are worth more than you realize, [NAME]. | SCAP2 True or False question:  ** Ignoring the haters and doubters can help you stay focused on your goals. ** Press 1 for true, Press 2 for false. Respond to the text to earn points and cash. | TRUE |
| 13 | Hi from SCAP2, [NAME]. Here’s your health text message for the week: One goal of taking HIV medication is to see your CD4 or T-cell count go up. | SCAP2 True or False question:  ** One goal of taking HIV medication is to see your CD4 or T-cell count go up. ** Press 1 for true, Press 2 for false. Respond to the text to earn points and cash. | TRUE |
| 14 | SCAP2 wants to know: Did you eat breakfast this morning, [NAME]?  Studies have shown that eating breakfast is linked to improved memory and concentration throughout the day. Here’s more info: [URL REDACTED] | SCAP2 True or False question: ** Eating breakfast is not linked to improved concentration throughout the day. ** Press 1 for true, Press 2 for false. Respond to the text to earn points and cash. | FALSE |
| 15 | We at SCAP2 know that stopping smoking is very difficult. But, if you smoke, nicotine patches, nasal sprays, and gums are a safer option than smoking cigarettes. Click the link for more information. [URL REDACTED] | SCAP2 True or False question:  ** Nicotine patches are just as harmful as smoking regular cigarettes. ** Press 1 for true, Press 2 for false. Respond to the text to earn points and cash. | FALSE |
| 16 | Hi, [NAME]! Note to self: This is your journey, your body, your mind, and your spirit. Dig deep, own it, and start doing things for you and by you. SCAP2 is thinking about you and sending support your way. | SCAP2 True or False question:  ** It is healthy to take charge of your mind, body, and spirit by taking time to do things that are just for you. **  Press 1 for true, Press 2 for false. Respond to the text to earn points and cash. | TRUE |
| 17 | We wish stress was not a problem for our SCAP2 participants, but we know it often is. Feeling overwhelmed? Try taking deep breaths. That can reduce blood pressure and anxiety. [URL REDACTED] | SCAP2 True or False question:  ** When you are feeling overwhelmed, taking deep breaths can help you relax. ** Press 1 for true, Press 2 for false. Respond to the text to earn points and cash. | TRUE |
| 18 | Hello from SCAP2, [NAME]. Need help remembering to take your medications? Using a pill box can help! Here are some additional tips to make taking medication easier. [URL REDACTED] | SCAP2 True or False question:  ** For most people, using a pill box will make it easier for them to remember to take their medications. ** Press 1 for true, Press 2 for false. Respond to the text to earn points and cash. | TRUE |
| 19 | It’s time for your SCAP2 health text message, [NAME]! Not ready to start HIV medication at this time? People can still benefit from going to a health care professional regularly. | SCAP2 True or False question:  ** People who choose not to take HIV medications can still benefit from attending regular health care appointments. ** Press 1 for true, Press 2 for false. Respond to the text to earn points and cash. | TRUE |
| 20 | Hello, [NAME]. A lot of people in our SCAP2 community are worried about HIV medication side effects.  Side effects are real, but today’s HIV medications are easier to take than ever and have fewer side effects than ever. Click for more info [URL REDACTED]. | SCAP2 True or False question:  ** Today’s HIV medications have fewer side effects than HIV medications in the past. ** Press 1 for true, Press 2 for false. Respond to the text to earn points and cash. | TRUE |
| 21 | Hi [NAME] from SCAP2. A lot of wise people think the secret of health for both mind and body is to not mourn for the past, not to worry about the future, or not to anticipate troubles, but to live in the present moment wisely and earnestly. | SCAP2 True or False question:  ** Focusing on the present, rather than on the past or the future can reduce stress and possibly improve your health. ** Press 1 for true, Press 2 for false. Respond to the text to earn points and cash. | TRUE |
